# Molecular mechanism of PARP inhibitor resistance

**DOI:** 10.18632/oncoscience.610

**Published:** 2024-09-23

**Authors:** Yi Huang, Simin Chen, Nan Yao, Shikai Lin, Junyi Zhang, Chengrui Xu, Chenxuan Wu, Guo Chen, Danyang Zhou

**Affiliations:** ^1^School of Biopharmacy, China Pharmaceutical University, Nanjing 211198, P.R. China; ^2^School of Public Health, Nanjing Medical University, Nanjing 210029, P.R. China; ^3^Department of Respiratory, Nanjing First Hospital, China Pharmaceutical University, Nanjing 210012, Jiangsu, P.R. China; ^*^Equal contribution

**Keywords:** PARP inhibitor, synthetic lethality, BRCA1/2, homologous recombination (HR) repair

## Abstract

Poly (ADP-ribose) polymerases (PARP) inhibitors (PARPi) are the first-approved anticancer drug designed to exploit synthetic lethality. PARPi selectively kill cancer cells with homologous recombination repair deficiency (HRD), as a result, PARPi are widely employed to treated *BRCA1/2*-mutant ovarian, breast, pancreatic and prostate cancers. Currently, four PARPi including Olaparib, Rucaparib, Niraparib, and Talazoparib have been developed and greatly improved clinical outcomes in cancer patients. However, accumulating evidences suggest that required or *de novo* resistance emerged. In this review, we discuss the molecular mechanisms leading to PARPi resistances and review the potential strategies to overcome PARPi resistance.

## INTRODUCTION

As genetic information, DNA is replicated continuously during an individual’s lifetime. Endogenous sources such as reactive oxygen species (ROS) and exogenous sources including heavy metals and genotoxic chemicals could damage DNA [1, 2]. DNA damage triggers a series of signaling cascades that promotes repair of broken DNA. Poly (ADP-ribose) polymerases (PARP) is a group of enzymes that catalyze ADP-ribose modification on the substrate proteins. PARP family contains 17 members and PARP1 is the most abundant one. As the DNA damage sensor, PARP1 recognizes and accumulates at damaged DNA sites, facilitating the recruitment of repair proteins through PARylation [3]. Inhibition of PARP1 results in DNA double-strand breaks (DSBs), which are the most severe type of DNA damage. PARP1 inhibition-induced DSBs are highly replies on homologous recombination (HR)-mediated pathway to repair. Consequently, PARP1 inhibition induces synthetic lethality in *BRCA1/2*-mutant cancer cells. Lynparza (Olaparib), the first PARP inhibitor (PARPi), was approved by the FDA in 2014 as a first-line maintenance treatment for BRCA-mutated advanced ovarian cancer. PARPi is the first approved-drug that was developed utilizing the concept of synthetic lethality. Unfortunately, a significant number of cases of acquired and *de novo* resistance to PARPi have emerged during treatment in clinics due to a series of complex interaction mechanisms. In our present review, we summarize the mechanisms for PARPi’s acquired and *de novo* resistance and propose therapeutic strategies to reverse resistance and optimize PARPi therapies in the future.

## DNA DOUBLE-STRAND BREAK REPAIR PATHWAYS

**Figure 1:**
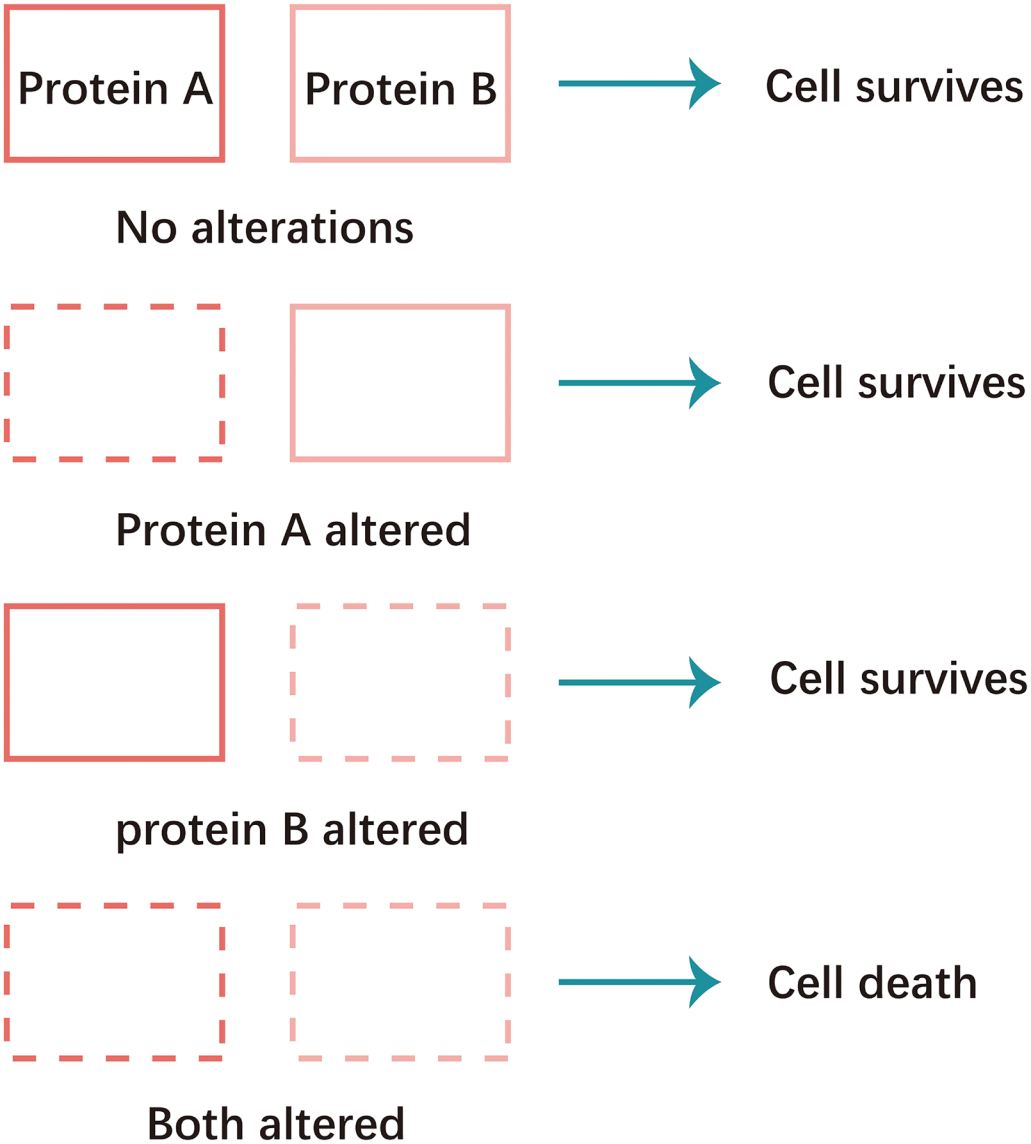
Schematic of synthetic lethality. Concomitant alteration of two genes or proteins results in cellular apoptosis, whereas altering either gene or protein individually does not elicit apoptotic response.

**Figure 2:**
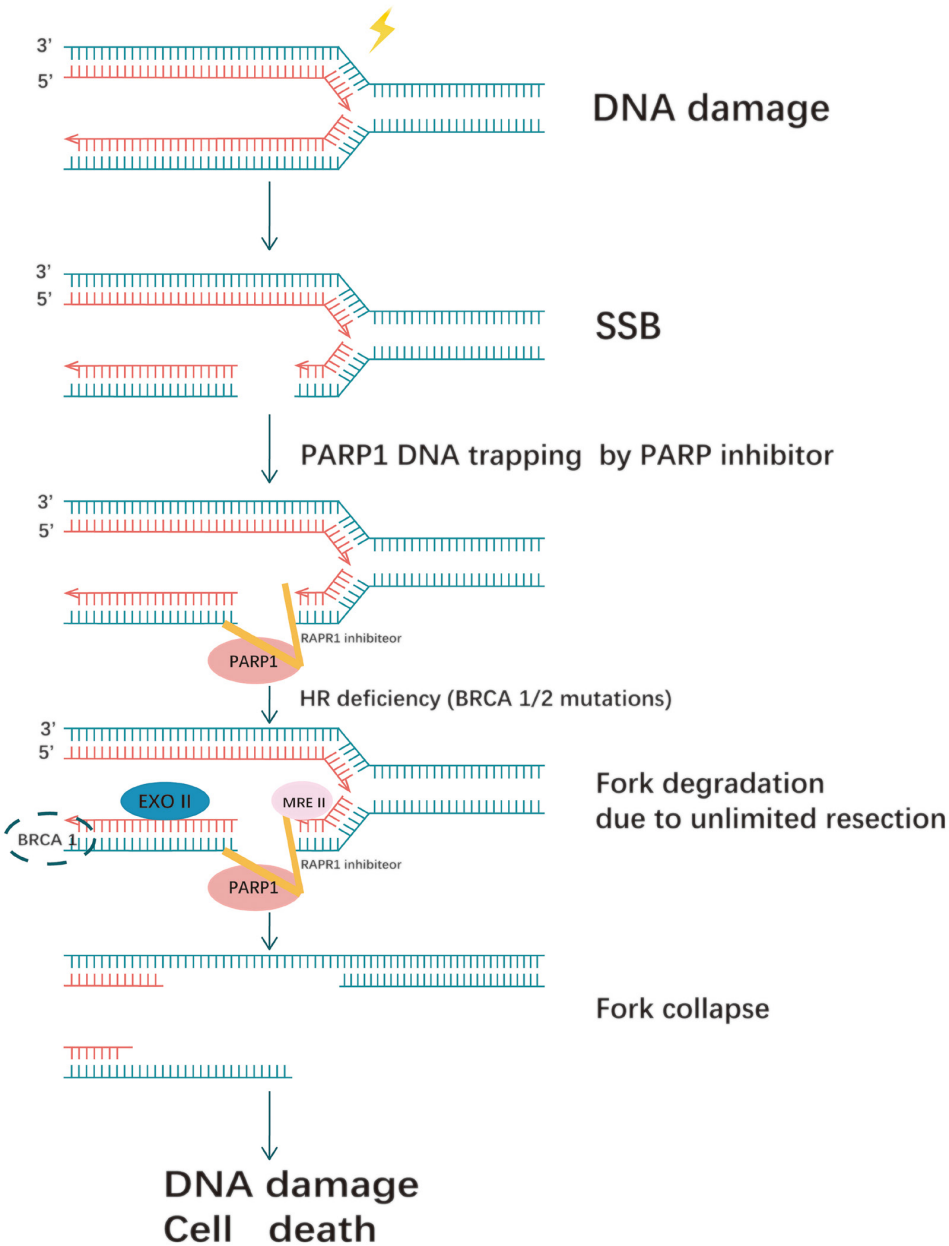
Synthetic lethality between PARP inhibitors and homologous recombination deficiency. PARP entrapment on DNA damage blocks the replication machinery, and loss of PARP activity prevents replication fork reinitiation. This results in DSBS requiring repair by homologous recombination. In the case of homologous recombination defects, such as BRCA1/2 mutations, PARP1-trapping lesions cause excessive fork degradation by the MRE11 nuclease, leading to fork collapse.

Non-homologous end joining (NHEJ) and homologous recombination (HR) are two major pathways to repair the DNA double- strand breaks (DSB). HR is a key pathway during late S phase to G2 phase of the mammalian cell cycle, as it leads to precise repair of DNA damage using the sister chromatid as the repair template, which is less error-prone and a more conservative path [4]. BRCA is a key protein in the HR pathway. NHEJ is an error prone pathway and is active throughout of the cell cycle. During NHEJ, DSB sites are repaired by blunt end ligation with low fidelity. Consequently, NHEJ usually results in small insertion or deletion. HR and NHEJ using different repair machinery to complete the repair process. For HR, DSBs are first processed by Mre11-Rad50-NBS1(MRN) complex mediated-end resection to produce 3′-ssDNA overhang, which is subsequently coated by RPA and finally displaced by Rad51 recombinase to complete the repair process. Regarding NHEJ, broken ends are firstly hold by heterodimer Ku70/80 (Ku) and then recruit DNA-PKcs, Artemis, XRCC4 and Ligase IV to fill the by error-prone DNA polymerases. Failure to repair DSBs leads to accumulation of genetic aberrations, cell cycle arrest and apoptosis. “Synthetic lethality” is a term used to describe a situation in which a defect in either gene has no or little effect on cell survival, but when both genes are defective together, the cell death rate is greatly increased [5] (Figure 1). The principle can be used to selectively kill tumor cells without affecting normal somatic cells. In BRCA1/2-deficient patients, inhibition of PARPs may lead to accumulation of DNA DSBs and resulting in cell death (Figure 2).

## PARP IN THE CONTEXT OF DNA REPAIR IN *BRCA1/2*-MUTATED CANCERS

### The mechanism of PARPi

**Figure 3:**
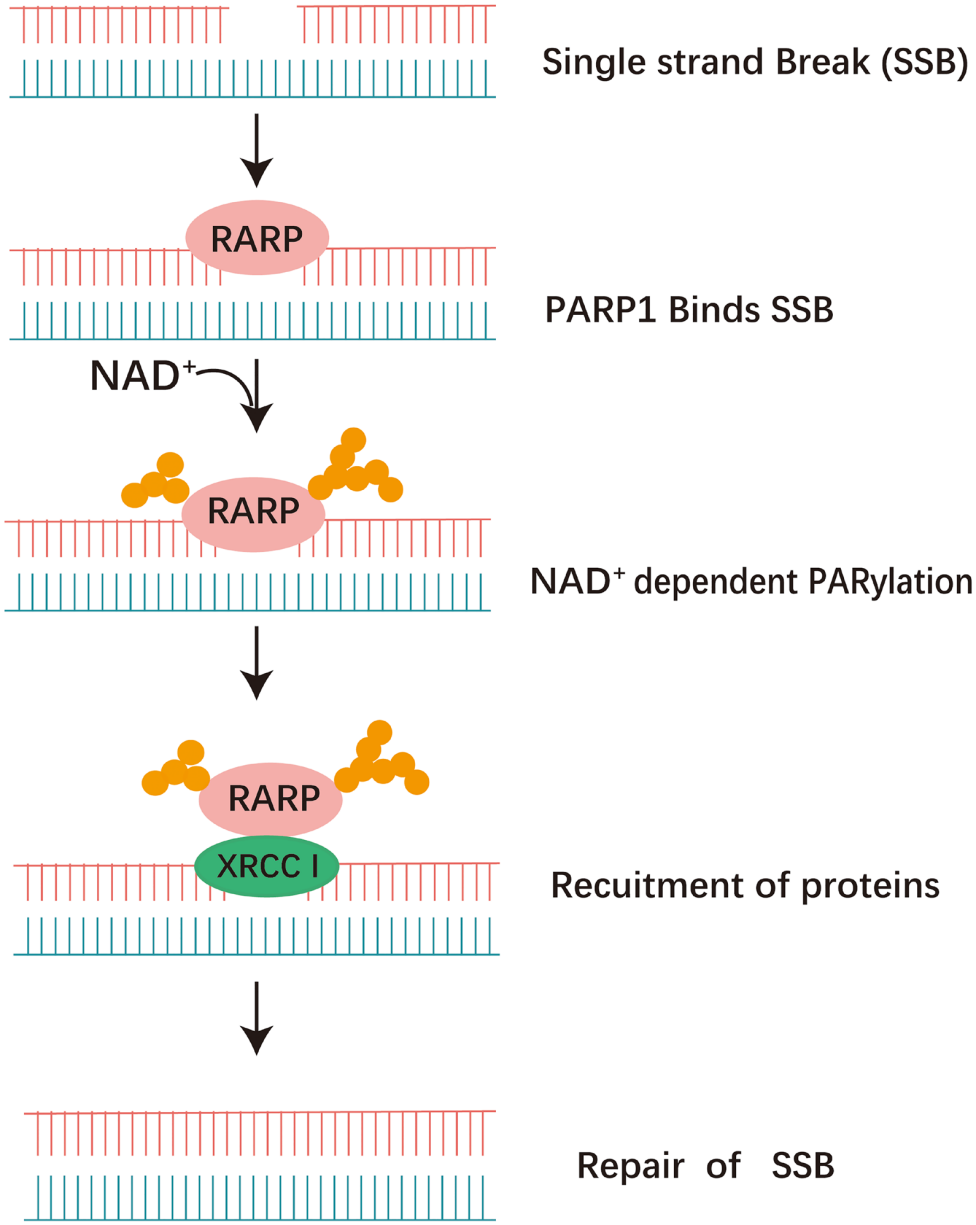
The activity of PARP1 in the repair of SSB via the BER pathway. DNA damage is quickly detected and bound by PARP. PARP utilizes NAD+ as a substrate to facilitate auto-poly(ADP-ribosyl)ation, form ADP-ribose polymers. this auto-modification recruits additional DNA repair molecules, such as XRCC1 to the site of damage, promoting the effective repair of DNA.

Poly (ADP-ribose) polymerases (PARP) inhibitors (PARPi) have been shown to be effective against homologous recombination repair deficient tumors in a synthetically lethal interaction. PARP1 is the primary poly (ADP-ribose) polymerase responsible for accelerating the global rate of single-strand break repair (SSBR) in human cells. PARP2 also has a role in SSBR and has an overlapping role with PARP1 for recruitment of XRCC1. In addition, PARP3 deficient cells also display genome instability and delayed repair of single strand break [6]. After DNA damage, PARP1 catalyzes the posttranslational polymerization of ADP-ribose units (PARs) from NAD+ molecules onto target proteins via covalent linkages to acidic residues. this auto- and hetero-modification recruits additional DNA repair molecules, such as XRCC1 to the site of damage, promoting the effective repair of DNA [7] (Figure 3).

PARPi are a class of anti-cancer drugs which compete with nicotinamide (NAD+) for the catalytically active site of PARP molecules. Two of the benzamides, 3-aminobenzamide and 3methoxybenzamide, were found to be competitive inhibitors, with K1 values of less than 2 μM [8]. As of the current moment, PARP inhibitors have been investigated either as standalone treatments in cancers with depleted BRCA1/2 genes or in cancers exhibiting BRCA-like characteristics. They are also being studied in combination with other DNA-damaging agents such as ionizing radiation across a wide spectrum of cancer types. A total of nine drugs are progressing through various phases of drug development [9, 10].

A large body of evidence has pointed to an involvement of PARP function in the base excision repair pathway, which generates SSBs as repair intermediates. PARP inhibition leads to persistent single-strand gaps in DNA [10]. DSBs can be produced by replication across a single-stranded nick or by rupture of a DNA strand at a stalled replication fork. If these gaps are encountered by a replication fork, arrest would occur and the single-strand gaps may degenerate into DSBs [11].

Two pathways dominate the repair of two- ended DSBs: NHEJ and HR. Normally these DSBs can be repaired by HR [12]. HR of an exchange type is induced by DSB associated with replication forks, HR at the HPRT gene is rapidly induced in the early S phase by DSB at replication forks [13]. Replication forks frequently stall during normal DNA replication at DNA lesions, or template-bound proteins [14]. Because of mechanisms that deal with stalled replication, stalling does not usually give rise to persistent DNA breakage, and BRCA2 is an essential component of corresponding mechanisms in mammalian cells. In the absence of BRCA1 or BRCA2, the replication fork cannot be restarted and collapses [15]. Resulting in persistent chromatid fragmentation that cannot be repaired. when HR is disrupted/altered, DSBs usually repaired by NHEJ are instead repaired by other mechanisms, that give rise to chromosomal translocations and deletions [16]. That would cause large numbers of chromatid breaks and aberrations, leading to loss of viability.

### Base excision repair

The PARP1 and PARP2 isoforms play a crucial role in the base excision repair (BER) pathway, particularly in addressing single-stranded DNA breaks. Constitutively present, these isoforms are activated in response to DNA damage. PARP1, upon activation, engages in the poly (ADP-ribosyl) ation of nuclear proteins. PARP1 comprises three domains, an N-terminal DNA binding domain (DBD), a central domain responsible for auto-modification (AMD), and a C-terminal catalytic domain (CD), accountable for DNA binding, self-modification, and enzymatic catalysis. The DNA-binding domain of the protein is characterized by two zinc finger structures and a nuclear localization sequence at the N-terminus. This domain is responsible for the recognition of both single-strand nicks (SSBs) and double-strand breaks (DSBs) in DNA [17]. PARP2 is another member of the PARP protein family closely associated with the PARP1 enzyme. It possesses a catalytic domain but lacks the N-terminal DNA binding domain [18]. PARP1 and PARP2 engage in mutual interactions and share common associates, including XRCC1, DNA polymerase beta, and DNA ligase III, which are directly implicated in the Base Excision Repair (BER) pathway [19].

When DNA damage occurs, the zinc-finger DNA-binding domains facilitate the recruitment of PARP1 and PARP2 to the damaged DNA site. Subsequently, PARP enzymes catalyze posttranslational modifications by adding ADP-ribose to specific nuclear proteins [18, 20]. PARP-1 interacts with SSB or DSB in DNA, facilitating the cleavage of poly (ADP-ribose) (PAR) from nicotinamide adenine dinucleotide (NAD+). This enzymatic process results in the release of nicotinamide and ADP-ribose as byproducts [21]. PARP utilizes NAD+ as a substrate to facilitate auto-poly (ADP-ribosyl) ation, as well as the poly(ADP-ribosyl)ation of other proteins, form ADP-ribose polymers.

PARP2 activity in response to DNA damage is about 10 times less than PARP1 activity, but PARP2 is also important, the PARP1/2 heterodimer have a concerted role during base excision repair [20]. Moderate activity of PARP1 is favorable for DNA repair while its overactivation might commit cells to death. PARP2 in regulation of PARP1 activity, to prevent overactivation of PARP1 bound to DNA damage [22].

**Figure 4:**
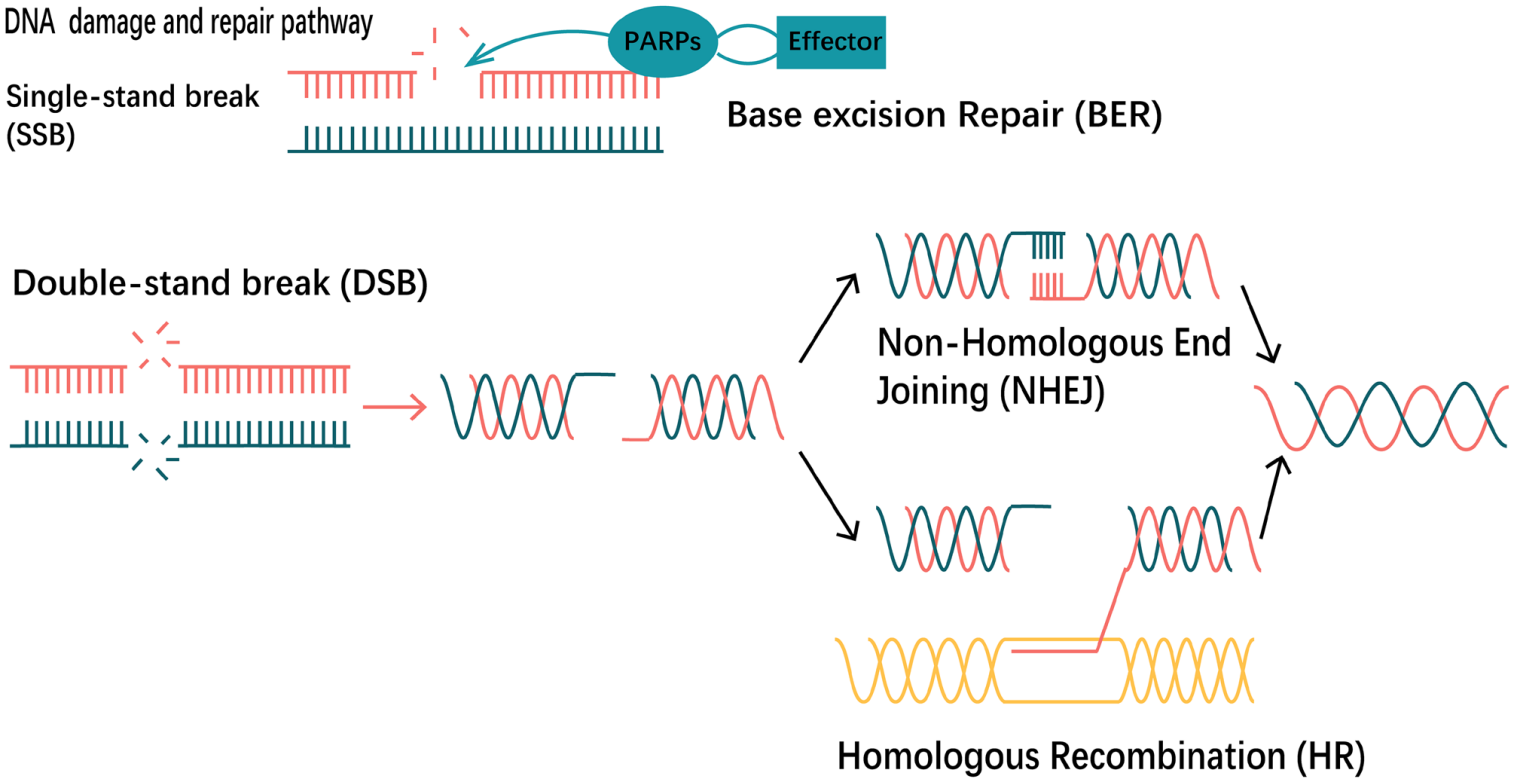
DNA damage and repair pathways. When a single strand break (SSB) occurs in DNA, PARPs and a series of effector molecules work together to repair the break, resulting in base excision repair (BER). When DNA undergoes double-strand break (DSB) damage, both non-homologous end joining (NHEJ) repair and homologous recombination (HR) repair pathways act together.

Long, branched, poly (ADP-ribose) (PAR) chains serve to attract the recruitment of the Base Excision Repair (BER) protein complex, facilitating the advancement of the repair process [23] (Figure 4). The PAR-Binding Motif features hydrophobic amino acids interspersed with charged basic residues [24]. PAR carries a negative charge, which enhances the enlistment of DNA repair proteins engaged in the Base Excision Repair (BER) pathway to the location of DNA damage, and assists in the displacement of PARP-1 and PARP-2 from the sites of damage, thereby enabling the entry of other repair proteins [25, 26]. Thus, PARP facilitates efficient DNA repair and survival of cells. Inhibition of PARP elevates the occurrence of DNA strand breaks, rendering PARP-deficient cells highly susceptible to carcinogenic agents [27].

### PARP1 trapping

Besides simply blocking PAR synthesis, PARPi may also kill tumor cells via a ‘trapping’ mechanism [28]. The subset of BER SSB intermediates that become uncoupled somewhere during the repair pathway, are bound by PARP1 when it is present in the cell. PARP inhibitors did not impair the recruitment of PARP-1 but block the reversal of this process, trap the PARP1 enzymes at damaged DNA [29], leading to the accumulation of significant amounts of PARP1 and PCNA at the sites of damage, thereby delaying their dissociation [30].

PARP activity can promote recruitment of other DNA repair proteins to accelerate repair of DNA strand breaks, of which XRCC1 and its protein partners are among the most important [31]. XRCC1 is a multidomain protein with no known catalytic activity. Yet, it interacts with a number of repair protein, e.g., PARP1, Pol b, and lig-IIIa, and is thought to function as a scaffold able to modulate and coordinate the various steps of BER. Poly (ADP-ribosyl) ation is mandatory for the recruitment of the XRCC1 protein by PARP-1 at sites of DNA damage [29], The trapping effect of PARP inhibitors inhibits this process.

In addition to the catalytic inhibition of PARP inhibitors, Trapped PARP-DNA complexes exhibit higher cytotoxicity compared to unrepaired SSBs resulting from PARP inactivation. This suggests that PARP inhibitor function, at least in part, as agents that trap PARP enzymes on DNA, acting as poisons. PARP inhibition by Olaparib is more cytotoxic than genetic depletion of PARP, it synergistically increases the cytotoxicity of MMS in a PARP1-dependent manner, as it induces the trapping of PARP1–DNA complexes [32].

PARP inhibitors potentiate the cytotoxicity of DNA alkylating agents such as methyl methanesulfonate (MMS) and temozolomide (TMZ) at least in part by preventing this destabilization, thereby trapping PARP1 at sites of DNA damage [33, 34]. PARP inhibitors trap PARP by 2 non-mutually exclusive mechanisms. One is by inhibition of PARylation, which increases the binding of PARP to DNA. It is related to the inhibition of catalytic activity; the other is drug binding to the NADþ site, which allosterically enhances the DNA binding of PARP’s N-terminal zinc finger domain for damaged DNA independent of catalytic inhibition [32]. Among them, the first mechanism whereby inhibition of automodification stabilizes DNA binding is more important [35].

**Figure 5:**
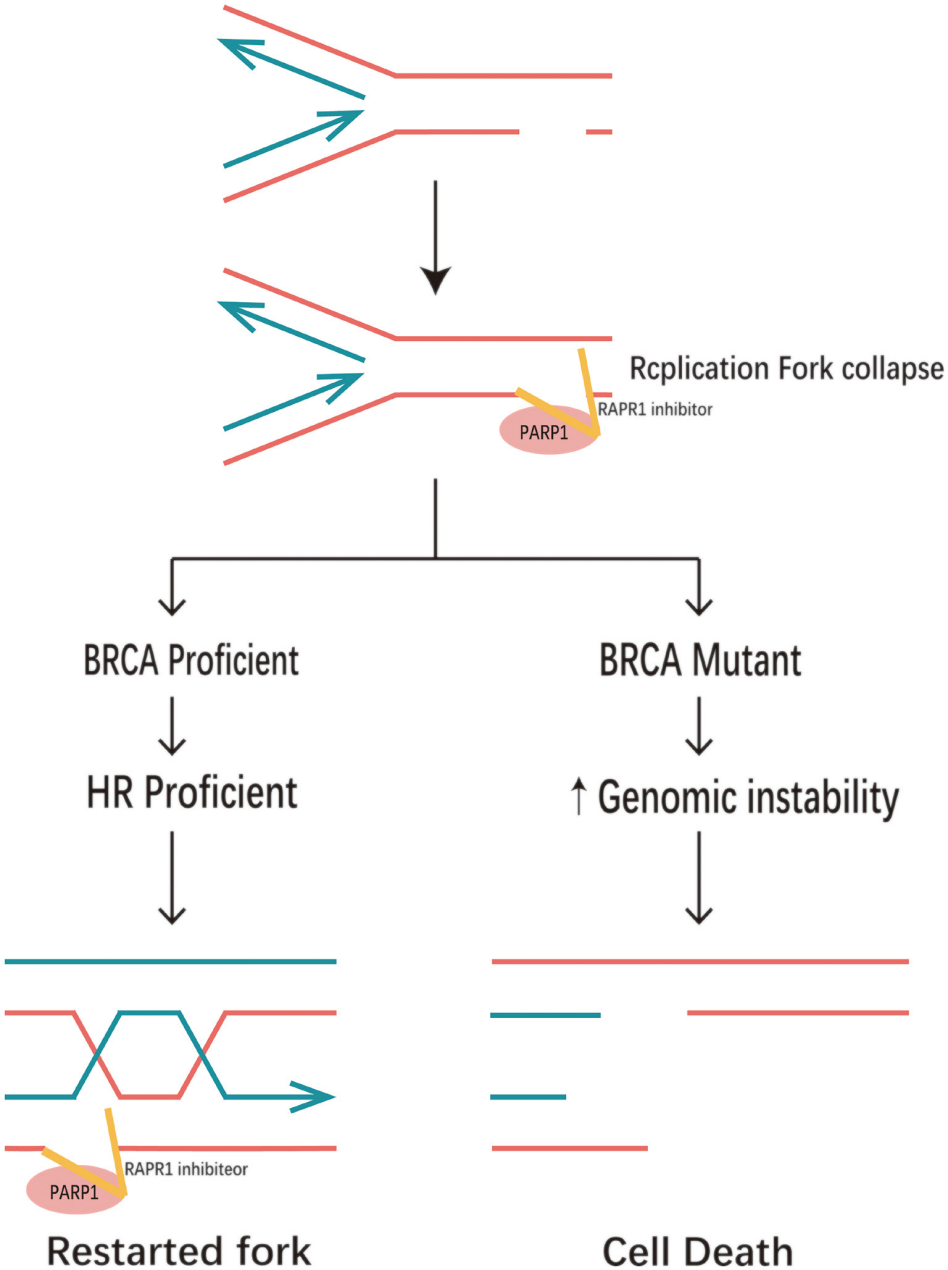
PARP trapping mechanism. PARP1 trapping lesions prevents PARP1 from leaving the site of DNA damage, blocking the replication machinery and leading to replication fork collapse. If BRCA is proficient, it can restart the replication fork: if BRCA is mutated, it can result in persistent chromatid fragmentation that cannot be repaired, eventually leading to cell death.

The single agent cytotoxicity of different PARP inhibitors does not correlate with their ability to inhibit PARylation [32, 33]. Hence, distinctions must be made between inhibitors based on their trapping potency, at least when considering single agent activity. Still, it is important to stress that all PARP inhibitors currently in the clinic are catalytic inhibitors, and where they vary is in their effectiveness in trapping PARP onto DNA [28] (Figure 5).

## THE MECHANISM FOR THE RESISTANCE OF PARPI

### The restoration of HR repair activity

**Figure 6:**
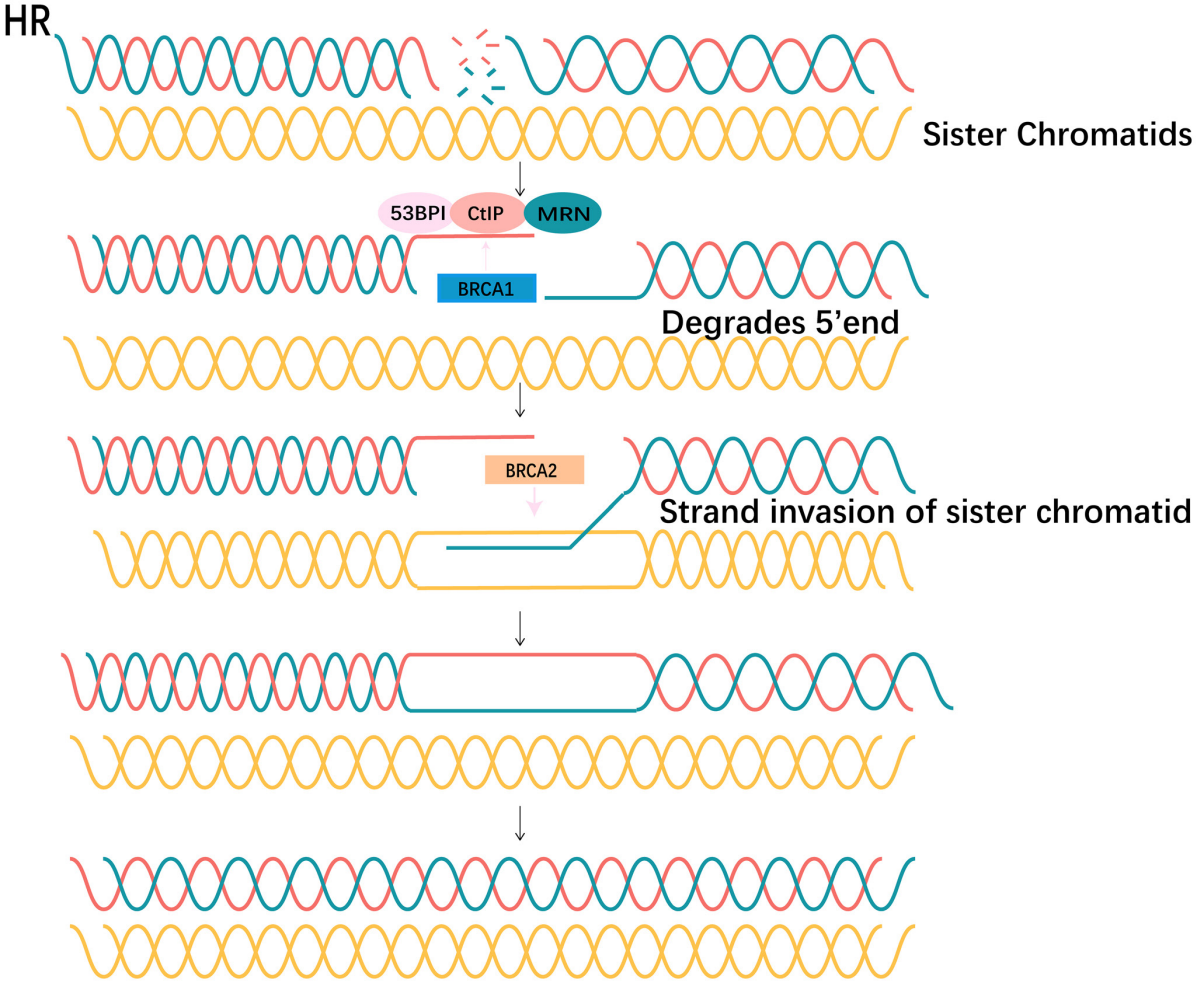
HR repair pathways. The DNA undergoes a DSB. BRCA1 and 53BP1 preempt binding sites at DSBs and together with factors such as MRN and CtIP affect base resection at the 5 ‘end. BRCA2 promotes the invasion of a strand of DNA into sister chromatids, which undergo homology pairing. Strands complete elongation and repair. DSB repair was completed.

As explained above, HR deficiency (HRD) is an important prerequisite for the synthetic lethality of PARPi. Therefore, restoration of HR activity would be expected to result in decreased sensitivity of cells to PARPi. According to the available experimental studies, there are several reasons for the recovery of HR (Figure 6).

### BRCA1/2 related

#### Reverse mutation of BRCA1/2

In HR repair, BRCA1/2 are key proteins involved in HR repair. BRCA1 promotes terminal resection to initiate HR, whereas BRCA2 mediates sister chromatid invasion. Loss of function due to wild-type BRCA genes in tumor cells is one of the causes of HR defects. In a large number of clinical reports, the recovery of BRCA1/2 function is the most common mechanism causing secondary PARPi resistance [36, 37], which occurs frequently and is highly correlated with the use of platinum-based drugs and PARPi [38]. BRCA1/2 gene revers mutation can recover the Open Reading Frame (ORF) of BRCA1/2 to restore protein expression [39, 40], and eventually lead to PARPi resistance in tumor cells through accumulation of cytogenetic mechanisms.

For BRCA1, it is composed of N-terminal domain (BRCT), N-terminal RING domain and coiled-coil domain. Among them, the conserved N-and C-terminal domains are critical for the response to targeted therapy in HR deficiency. Thus, BRCA1-deficient cells with mutations that disrupt either the N - or C-terminal domains have a poor response to PARPi [38]. For example, in BRCA1-deficient breast cancer mice, a model carrying the BRCA1C61G missence mutation were found to have broken the N-terminal RING domain of BRCA1, and be significantly resistant to PARPi [41]. BRCA2 contains a DNA-binding region and BRC repeats. And BRC plays an important role in RAD51 binding and mediation of the recruitments of RAD51 and strand exchange in HR. BRCA2-deficient ovarian cancer cells carrying a frameshift mutation in the allele c.6174delT have been found BRC-altered and confirmed to be PARPi resistant [42]. A secondary BRCA2 c.9106C>G mutation was identified in the DNA of patients with PARPi resistance, resulting in substitution of the stop codon with another amino acid and restoration of BRCA2 function [39]. In addition, patients presenting with the secondary BRCA2 mutation c.4705_4708delGAAA also have PARPi resistance [39]. A series of recurrent mutations affect the key functional sites of BRCA1/2 by deleting amino acid residues, adjusting the number of amino acids, changing the position of stop codons or other ways, and restoring the ORF. However, the data show different proportions of BRCA1/2 reversal mutations in different cancer patients, and further validation is needed in a larger base of samples [43, 44].

#### Reversal of BRCA1 promoter methylation

In some tumor samples, BRCA1 promoter methylation caused a decrease in BRCA1 expression, resulting in a loss of BRCA1 function. Although BRCA1 promoter methylation has no obvious effect on tumor development per se, it can increase the sensitivity of tumor cells to PARPi [45]. Therefore, mechanistically, achieving BRCA1 promoter demethylation in these cells also leads to PARPi resistance. Actually, BRCA1 can indeed be restored to normal expression levels by demethylation when detected in clinical tumor recurrence samples [38]. What’s more, it has been confirmed that the repair of BRCA1 hypermethylated promoter is also one of the repair pathways of HRR in patient-derived xenograft (PDX). However, the exact mechanism of BRCA1 demethylation is still unclear.

In addition to BRCA1/2 reverse mutations and promoter demethylation, HR restoration by BRCA1/2-independent mechanisms was also found in a large proportion of BRCA1/2 deficient cells, which was confirmed to cause PARPi resistance in mouse tumors [46].

### DNA end resection

NHEJ is divided into classical non-homologous end joining (C-NHEJ) and alternative non-homologous end joining (A-NHEJ) that including the more common microhomology-mediated end joining (MMEJ) involving the DNA polymerase POLQ. Among them, NHEJ is a non-template repair, which easily leads to gene errors, aberrations, and chromosome instability. HR, on the other hand, is more critical because it uses the complementary DNA strand of the adjacent sister chromatid as a template with higher accuracy.

The choice of the two pathways is biased in different cell cycles, which is related to the way of DNA end resection. The different degree of DNA end resection determines the different overhang length of 3 ‘single-stranded DNA (ssDNA), which is a primary factor affecting the selection of DNA damage repair pathway [47]. C-NEHJ does not require DNA end hanging and can occur throughout the cell cycle and, in particular, is more prominent in the G0 and G1 phases. In contrast, MMEJ requires minimal end resection to produce short microhomology fragments (<25 nucleotides) and shows an advantage in early S phase [47, 48]. HR requires full exposure of the 3 ‘end of DNA to invade the homologous region and ensure the accuracy of repair, which occurs in S and G2 phases [48]. For example, MMEJ is activated by local DNA end resection, where the MRE11-RAD50-NBS1 (MRN) complex and C-terminal binding protein interacting protein (CtIP) introduce gaps near DSB lesions and degrade DNA using 3 ′–5′ exonuclease activity, whereas more extensive resection by BLM/ Exo1-catalyzed is more effective for HR [48]. Meanwhile, REV7, PTIP, RIF1 and other proteins have been observed to block the occurrence of HR by inhibiting DNA end resection, and inversely promote NHEJ selection [38].

Understanding the factors that influence HR and NHEJ pathway selection is critical to understanding PARPi resistance: When DNA damage occurs, 53BP1 recognizes and binds DSBs, and recruits a range of DNA damage repair factors. Moreover, 53BP1 has a positive effect on c-NHEJ by cooperating with downstream products, such as RIF1-Shieldin complex and PTIP, to limit DNA end resection [47]. However, BRCA1 crowded out 53BP1 binding sites when occupying DSBS, and together with CtIP, MRN, promoted extensive DNA resection (Figure 6). In addition, three nucleases MRE11, EXO1 and DNA2/BLM play important roles in the process of DNA end resection. Loss of the excision pathway responsible for all three proteins results in profound excision defects and HR pathway blockage [47].

The current study suggests that the expression of several of the above key proteins that influence the selection of DNA damage repair pathways, even in the absence of BRCA1/2, can influence PARPi resistance to some extent by modulating the dependence of cells on HR repair.

- CtIP protein is a key factor for DSB repair. The study found that, Spleen associated tyrosine (Syk) overexpression in high-grade ovarian cancer (HGSOC) and triple-negative breast cancer (TNBC) enhanced the ability of CtIP to ablate DNA ends after adequate phosphorylation, leading to HRR restoration and PARPi resistance [49].
- MRN *In vitro* experiments have shown that mutations in the MRN complex lead to increased sensitivity of tumor cells to PARPi. For example, knockdown of MRE11, one of the components of the MRN complex, resulted in increased sensitivity of tumor cells to PARPi [50, 51]. Conversely, overexpression of MRN complex may contribute to PARPi resistance. Moreover, histone acetyltransferase PCAF recruits MRE11 and EXO1 to acetylate H4K8, which aids in replication fork degradation in BRCA-deficient cells. In certain BRCA2-mutated cancers, decreased PCAF stabilizes stalled forks and confers resistance to PARPi [52].
- 53BP1 Loss of 53BP1 reversed HR loss and reduced PARPi sensitivity in BRCA1-deficient cells, but had no significant effect in BRCA2-deficient cells [53, 54]. Meanwhile, downstream compounds that cooperate with 53BP1 also play a role in PARPi sensitivity. Among them, REV7, as part of the Shieldin complex, is a key protein that coordinates the selection of cellular DNA double-strand break repair pathways [55]. In cellular molecular analysis, the Shieldin complex is a downstream effector of 53BP1-RIF1, which inhibits 5 ′–3′ DNA end resection by binding to ssDNA and recruiting cell signaling technology (CST) complex. The ssDNA overhangs required for HR repair be cut off, causing the cells to turn to NHEJ [55]. Thus, loss of REV7 restores DNA end resection by CtIP at DSBs and HR pathways, leading to PARPi resistance [53, 56]. Restoration of HR and PARPi resistance were confirmed by reconstructing DSBs end resection in Rev7-null human and mouse BRCA1-deficient cells [57].
- POLQ DNA polymerase theta (POLQ) is a key protein mediating MMEJ and prevents overexposure of ssDNA gaps. One study showed that high expression of POLQ was found in BRCA2-deficient ovarian cancer tumor cells, and it was suspected that the elevation of the alternative pathway MMEJ complemented the HR defect. The genomic instability of cancer cells and their cellular hyper-reliance on alternative poly-ADP ribose polymerase (PARP)-mediated DNA repair pathways are explained by the loss of HR [58]. In past reports, the combination of POLQ inhibitors (POLQi) and PARPi has shown great potential in the treatment of patients with BRCA-deficient tumors [59].

In addition to the changes in the above proteins that directly cause PARPi resistance, research data suggest that the related protein functional activity factors also mediately regulate the sensitivity of cells to PARPi, and indeed can lead to PARPi resistance. For example, cyclin-dependent kinases (CDKs) promote end resection by mediating the phosphorylation of MRN complexes and CtIP. Numerous cell assays and clinical cases have shown that the ability of CDKs to block end resection induces PARPi resistance [60–63]. Besides, inhibitor of DNA-binding 3 (ID3) [64], bromodomain-containing protein 7 (BRD7) [65], identified inositol polyphosphate 4-phosphatase type B (INPP4B) [66], mediator of DNA damage checkpoint protein 1 (MDC1) [67], Polo-like kinase 1 (Plk1) [68], Zinc finger432 (ZNF432) [69], salt-inducible kinase 2 (SIK2) [70], DNA helicase B (HELB) [71] and so on. It has also been shown that the chromatin environment and the complexity of DSB end also affect the selection of DNA damage repair pathways [47]. At present, the influence of these reasons on PARPi resistance cannot be completely denied.

At this time, HRR defects caused by BRCA1/2 deletion lose importance, HRR is restored under the action of other factors, and the efficiency of synthetic lethality is greatly reduced, which leads to PARPi resistance.

## MUTATION OR DELETION OF PARP

When DNA undergoes single-strand break (SSB), base excision repair (BER) is activated. PARPs signaling responds rapidly [72], recruiting DNA repair factors such as MRE11, EXO1, BRCA1, and BRCA2, and poly (ADP-ribosylation) (PARylation) occurs [73]. PARylation is a reversible modification process mediated by PARP to form poly (ADP-ribosylation) chain (PAR). PARP1 is the master protein in PARylation in most cells [73–75], which recognizes sites of DNA damage through zinc finger proteins [76]. PARPi can kill tumor cells by inhibiting the PARP1 enzyme and trapping the PARP1-DNA complex to impedes ssDNA break repair [57, 77]. Therefore, when PARP1 is mutated or its expression is reduced, PARPi loses its specific binding target, leading to PARPi resistance.

Theoretically, reduced PARP1 expression decreases PARPi sensitivity by reducing PARPi binding sites, as has been shown in the PDX model of breast cancer, and *in vitro* studies in human ovarian cancer tumor lines have shown that PARP1 deficiency leads to PARPi acquired resistance [37, 57, 78]. However, resistance induced by PARP1 knockdown is uncommon in clinical practice [79].

Besides, the loss of function caused by PARP mutations is also one of the causes of PARPi resistance. Part of the PARPi-resistance population lacked the nucleotides encoding amino acid residues K119 and S120. Therefore, ZnF domain mutations caused the reduction PARP1-DNA binding efficiency and resulted in PARPi resistance. In addition, part of PARPi resistance is attributed to mutations in amino acid residues which interact with hydrogen bonds connecting the DNA binding domain and catalytic domain, such as D45, H742, D743 E688, leading to PARPi resistance by disrupting PARP1 capture [80]. Actually, *de novo* PARPi resistance caused by PARP1 mutation had been identified in ovarian cancer patient [80].

Furthermore, PARG is the catalyst of the reverse reaction of PARylation and catalyzes the decomposition of PAR chains [81]. The reduction of PARG expression increases the efficiency of PARylation to a certain extent, resulting in more accumulation of PAR chains, which theoretically hinders the PARPi pathway due to the reduction of PARP1-DNA binding [82]. Genetic screening has confirmed that PARG deficiency induced PARPi resistance in mouse breast tumor cells, and even this mechanism was relatively common in BRCA2-deficient mouse breast tumor cell resistance [46]. Therefore, the search for factors that can induce PARG overexpression may be helpful in patients with drug resistance due to PARG loss.

## REPLICATION FORK STABILIZATION

Recent studies have shown that replication fork stability also makes BRCA gene-deficient cells chemoresistant through a series of complex interaction mechanisms [83]. When DNA replication hits a roadblock including exogenous and endogenous sources of genotoxic stress [84], which slow, stall, collapse, and break DNA replication forks, cells will respond to replication stress through fork reversal, fork breakage or fixing or bypassing DNA damage to maintain genome stability [85].

### Loss of replication fork reversal

Replication fork reversal is a dual-edged sword, it allows cells time to repair or bypass DNA lesions, but it is deleterious if left unprotected [85]. However, if the cell is unable to undergo replication fork reversal, the cell will not have the chance to produce enzymatically degraded substrates, or to undergo subsequent replication fork repair or bypass DNA lesions, which would have theoretically increased the replication fork’s stability, and decreased stress-induced DNA breaks in BRCA-deficient cells and increased resistance to PARP inhibitors [86].

**Figure 7:**
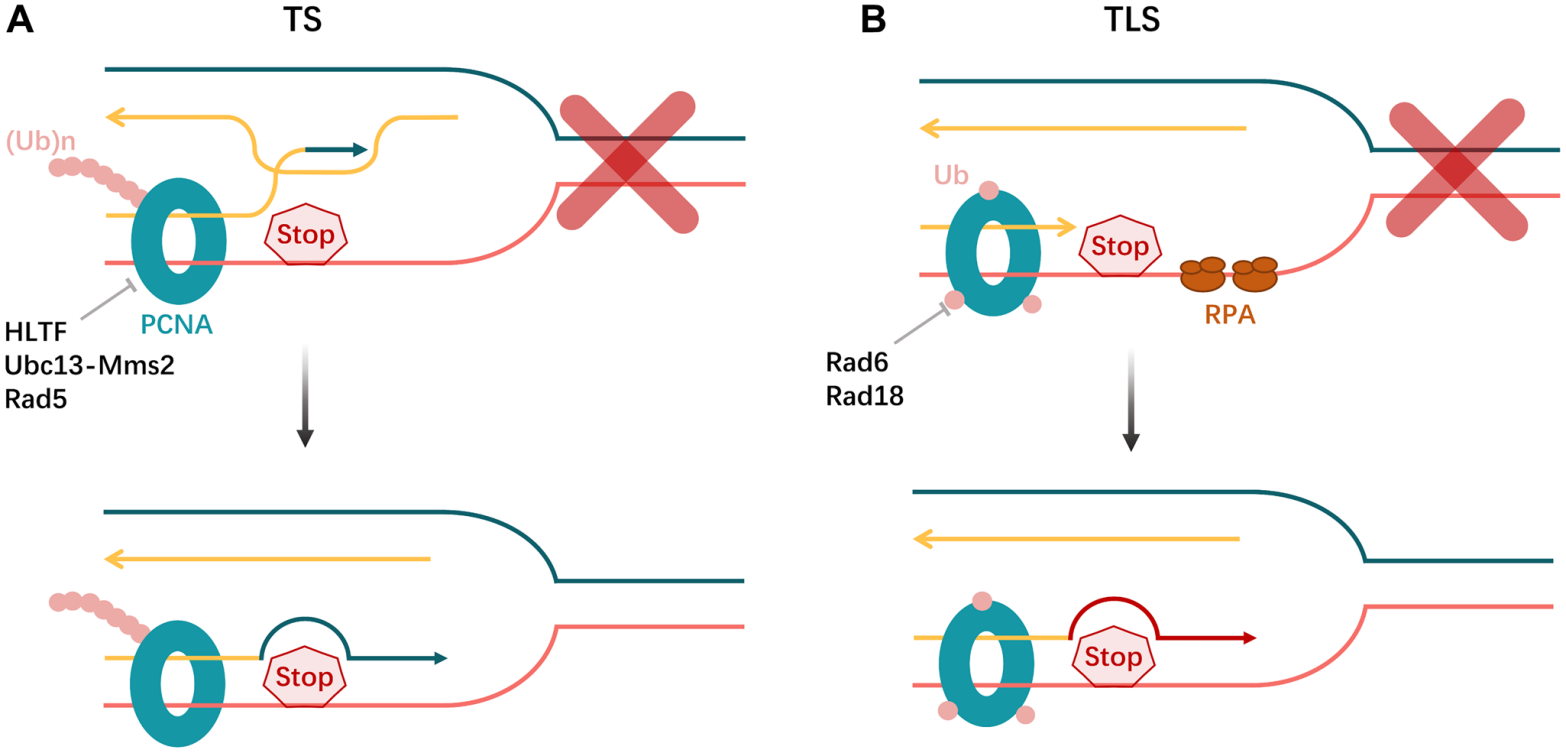
Cells employ post-replicative repair to filling the DNA replication gaps. (**A**) Template switching (TS) is triggered by poly-ubiquitylation of PCNA and mediated by HLTF, Ubc13-Mns2 and Rad5. (**B**) Replication protein A (RPA) also Poly-ubiquitylation of PCNA promotes translesion DNA synthesis (TLS) and is mediated by Rad6 and Rad18.

**Figure 8:**
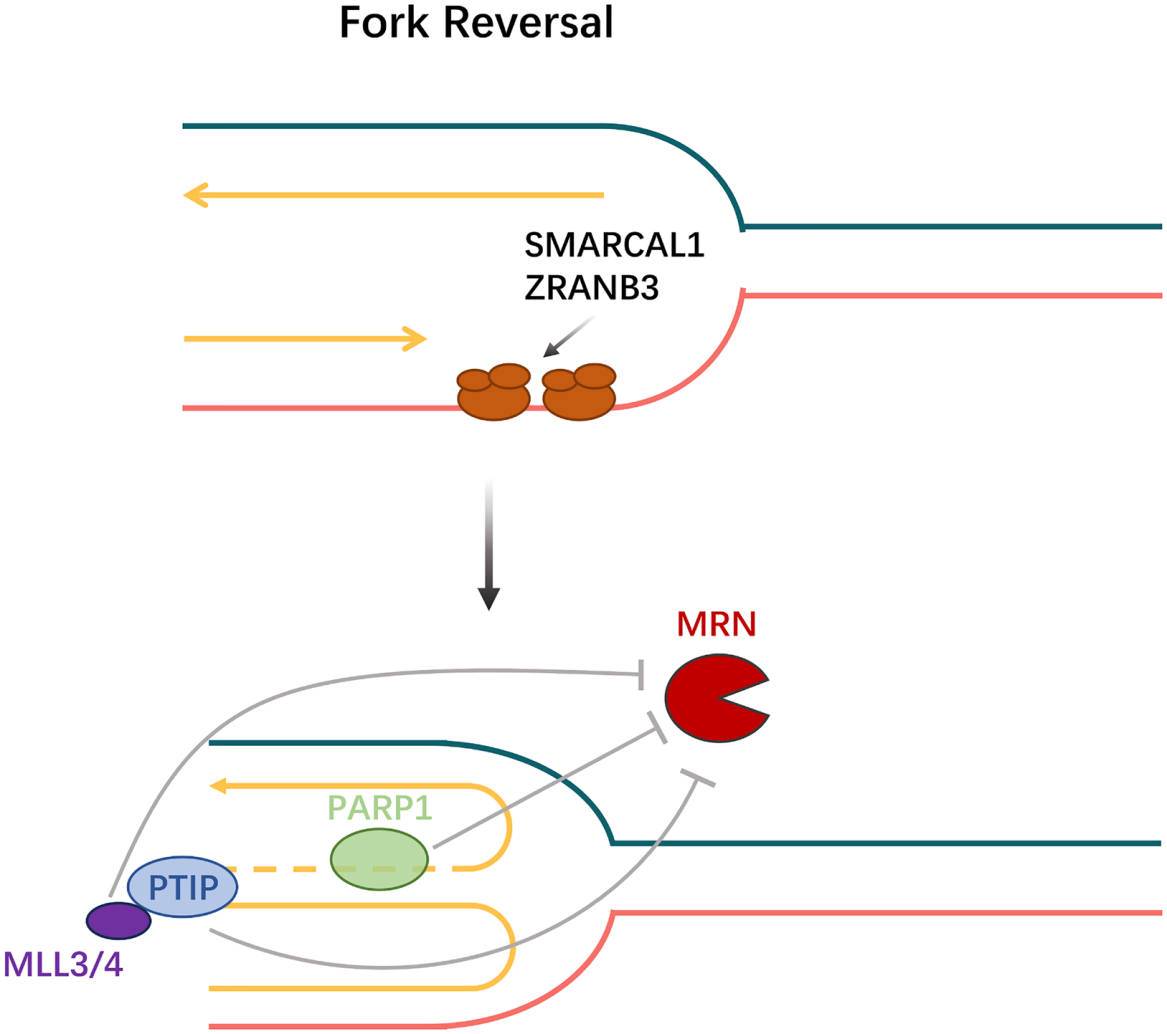
Fork reversal. SMARCAL1 and ZRANB3 recruited to stalled forks by RPA-bound ssDNA promote fork remodeling to facilitate fork reversal. MLL3/4 influence the function of MRN through MRE11 nuclease recruitment to stopped replication forks together with PTIP and mediating chromatin opening.

Members of the SNF2-family, SMARCAL1, ZRANB3, and HLTF, promote fork remodeling to facilitate fork reversal [87] (Figure 7A). The polyubiquitinated form of the DNA polymerase clamp PCNA, which is mediated by HLTF and possesses both ubiquitin ligase and fork-remodeling activities, recruits ZRANB3 to replication fork stalling sites [87]. SMARCAL1 is recruited to stalled forks by RPA-bound ssDNA [86] (Figures 7B and 8). Additionally, recent studies have revealed that breast cancer cells lacking BRCA1 and SMARCAL1 exhibit resistance to PARP inhibitors and cisplatin, which is accompanied by RF stabilization but does not result in HR restoration [86, 87]. Furthermore, the two remaining SNF2 family genes are unable to compensate for the loss of any one of the aforementioned SNF2 family genes, which results in PARP inhibitors resistance [86].

### Replication fork protection from degradation

MRE11, recruited at stalled replication forks, is implicated in ssDNA degradation with endonuclease activity that promotes 5 ′–3′ resection of DNA ends [88], and contributes to the instability of nascent DNA as a crucial factor [89]. BRCA2 is crucial in preventing MRE11-dependent degradation of reversed replication forks [85]. When PARP inhibitor is administered to BRCA-deficient cells to generate replication stress, MRE11 exonuclease shows increased activity, leading to significant degradation of the cells’ nascent DNA strand and promoting genomic instability [83].

Nevertheless, not all BRCA-deficient cells exhibit an elevated response to PARP inhibitors, and a subpopulation of cells remains immune to DNA damage because of deficiencies in the PTIP (Pax interaction with transcription-activation domain protein-1) or MLL3/4 histone methyltransferase complex [83, 90, 91]. At BRCA1/2-deficient double-strand breaks, HR activity is not restored by PTIP absence. Rather, its lack prevents the MRE11 nuclease from being drawn to replication forks that have stalled, so shielding developing DNA strands from severe destruction [83] (Figure 8). Furthermore, there is a connection between MLL3-mediated chromatin opening and MRE11 nuclease recruitment to stopped replication forks [92]. For this reason, chromosomal aberrations have partially recovered in BRCA2/MLL4 doubly deficient cells [83]. A genome-wide shRNA screen showed that BRCA2 cells exhibit chemoresistance with deletion of the nucleosome remodeling protein CHD4, and that resistance recovery is not dependent on HR but is linked to increased tolerance to DNA damage [93]. Along with MRE11, PARP1 participates in the stalling and restarting of the replication fork and facilitates MRE11’s localization during the fork stall as well as the formation of effective ssDNAs [94]. Hence, whereas PARP inhibitors may subject BRCA-deficient cells to genotoxic stress, PARP1 deletion somewhat preserves the cells’ genetic integrity [83]. Based on the evidence available, it appears that acquired resistance to PARP inhibitors could be primarily caused by replication fork protection.

## DNA REPLICATION GAPS SUPPRESSION

According to recent research, PARP1 regulates the rate of fork elongation and recognizes the unligated Okazaki fragments (OF), both of which are critical functions in DNA replication [95]. The BRCA protein fails to close replication gaps in cells with a defective BRCA-mediated HR pathway [96]. Meanwhile, through its ability to synthesize protein-conjugated polymers of ADP-ribose or PAR, PARP1 attracts proteins to ssDNA [96]. PARP1 can capture the ssDNA to assist other effectors to fill the DNA replication gaps. Thus, DNA replication gaps are a critical factor in determining the synthetic lethality of PARP inhibitors in cases of BRCA deficiency [96]. However, the cells in a patient with Fanconi anemia who had one mutant RAD51 allele were shown to have a growing ssDNA and to be sensitive to PARP inhibitors even though they had a functioning HR pathway. This indicated that the ssDNA persistence was what caused PARP inhibitor sensitivity, independent of the HR pathway condition [86].

Replication gap suppression (RGS) on the lagging strand, declining the unligated Okazaki fragments, lessens the production of S-phase poly-ADP-ribose (PAR), and several other effectors, excluding BRCA and PARP, are expressed in BRCA-deficient cells to finish OF processing, which inhibits PARP function resulting in to resistance to PARP inhibitors [86, 97]. In another way, the unreplicated ssDNA gaps on the leading strand, however, are the result of human Primase and DNA-directed Polymerase (PRIMPOL)-regulated fork repriming skipping and will be filled in following replication by translesion synthesis (TLS) or template switching (TS) termed post-replicative repair (PRR) [98] (Figure 7). Theoretically, all of the aforementioned ssDNA gaps in BRCA-deficient cells, brought on by unligated Okazaki fragments or PRIMPOL repriming, will eventually increase PARPi sensitivity because they will accumulate and cause genomic instability without BRCA1/2-mediated HR filling the DNA replication gaps [86]. In addition to the above-mentioned reduction of PAR in S-phase and restoration of OF processing on the lagging strand, other factors that support genomic stability and PARP inhibitor resistance include an increase in replication protein A (RPA) levels and TLS- or TS-mediated PRR enhancement on the leading strand [86]. The two primary sub-pathways of DNA damage tolerance (DDT) are error-prone TLS and error-free TS, which are facilitated by the sliding clamp proliferating cell nuclear antigen (PCNA) and are stimulated by PCNA polyubiquitination mediated by Ubc13-Mms2/Rad5 in late S-phase and PCNA monoubiquitination reliant on Rad6/Rad18 in G2-phase [99]. Furthermore, in BRCA-deficient cells, it has been discovered that MED12 deletion restores HR and increases replication fork stability, which results in RGS and PARP inhibitor resistance.

## THE ARISING OF ALTERNATIVE FACTORS IN DSB REPAIR PATHWAYS

Many additional DSB repair pathways, including as Nonhomologous End Joining (NHEJ) and Alternative end joining pathways (Alt-EJ) including microhomology-mediated end joining (MMEJ) also termed theta-mediated end joining (TMEJ), can replace HR in DSB repair to exhibit PARP inhibitor *de novo* resistance through improving genomic stability, even if BRCA-mutant cells have a defective HRR and cannot repair fatal DSB [100]. DNA polymerase theta (Polθ), which is mostly in charge of mediating TMEJ, is encoded by the POLQ gene. According to a recent study, BRCA-mutant breast cancers with a defective HRR showed increased POLQ expression. It exactly demonstrates that in contrast to normal, Polθ-mediated TMEJ repair arises as an alternative repair route and interacts with synthetic lethal genes for HR repair in tumors of the BRCA-deficient phenotype [101]. Thus, DSB-repair-deficiency depends on Polθ mediated-TMEJ, resulting in PARP inhibitor *de novo* resistance when Polθ is overexpressed.

## EPIGENETIC MODIFICATION

Epigenetic modification refers to the regulation of gene expression, through chemical modifications to alter DNA and proteins on chromosomes, thereby affecting gene expression. This modification can affect multiple levels of gene transcription, splicing, stability, translation, nucleosome assembly and chromatin structure, thus affecting the physiological and pathological processes of cells, as well as the phenotype of offspring. Through epigenetic changes, particularly abnormal modification of histone proteins or genomic DNA, tumor cells develop significant resistance to PARP inhibitors.

### BRCA1 promoter methylation

Impaired HR was usually detected in neoplasm samples, which is related to BRCA1 epigenetic silencing. Normally, reduced methylation levels in the BRCA1 promoter region show an epigenetic silencing effect and reduced BRCA1 mRNA expression levels [102]. BRCA1 knockdown increased sensitivity to PARP [103]. But a new mechanism for *de novo* resistance in relation to restoration of HR through BRCA1 promoter methylation was detected. Place BRCA1 under the transcriptional control of a heterologous promoter so that BRCA1 can re-express, but BRCA1 promoter hypermethylation is retained [104]. The specific mechanism is shown that the first base of BRCA1 exon 2 was fused to other base, and full-length BRCA1 protein was detected in resistant tumors by immunoblot analysis [104].

In addition, there are additional consequences of BRCA1 inactivation in myeloid neoplastic cells that give transformed cells a growth advantage. BRCA1 recruits a repressive complex to directly inhibits the promotor of miR-155. miR-155 is an overexpressed oncomiR, which has been proved to promote myeloid lineage expansion of hematopoietic stem cells [104]. So, the silencing of BRCA1 could be related to increased miR-155 levels, causing myeloid malignancies.

### Acetylation of 53BP1

p53-binding protein 1 (53BP1) is a large scaffold protein that mediates interactions with modified histones and several effector proteins and consists of multiple interacting surfaces of Double Strand Breaks (DSB) reactions.

53BP1 avoids mutation-repair outcomes by controlling the processing of DNA ends and dynamical evolution of DSBs. Studies have shown that 53BP1 plays an important and multifaceted role in Non- Homologous End Joining (NHEJ) mediated DSB repair. The specific mechanism is that 53BP1 is recruited to the chromatin structure around the DSB site and promotes NHEJ repair, and TIRR is the regulatory molecule of this recruitment process [105]. The primary function of TIRR is to bind to the tandem Tudor domain of 53BP1 when there is no DNA damage, masking its H4K20Me2-binding motif and thus keeping 53BP1 away from chromatin [105]. At the same time, 53BP1 also blocks DNA end-resection-dependent HR, which determines DSB repair pathways.

Acetylation of 53BP1 has been proved to inhibit NHEJ by negatively modulating recruitment of 53BP1 and promotes HR to decrease PARP inhibitors sensitivity in BRCA1-deficient cells. The mechanism is shown that the acetylation of K1626/1628 in the CPB-mediated UDR mediated disrupts the interaction between 53BP1 and the nucleosome, and subsequently blocks the recruitment of 53BP1 and its downstream factors PTIP and RIF1 [106].

Hyperacetylation of 53BP1, similar to the absence of 53BP1. The loss of 53BP1 similarly made BRCA1-mutated cells resistant to PARP inhibition and restored error-free repair of HR. Mechanistically, the loss of 53BP1 promotes ATM-dependent processing of the ends of broken DNA to produce recombinant single-stranded DNA [107]. More importantly, 53BP1 is a candidate predictive biomarker for PARPi response [108].

### PARP1 phosphorylation

PARP inhibitors kill tumor cells by inhibiting the activity of PARP enzymes and trapping. Therefore, the main reason for the resistance of tumor cells to PARP inhibitors is the increase of PARP activity and the recovery of PARP. In the screening of resistance to PARP inhibitors using genetic screening method of highly active piggyBac transposon in haploid mammalian cells, it was found that the toxicity of PARP inhibitors to normal cells was related to the expression of PARP1. the specific performance is that cells with low expression of PARP1 and cells carrying PARP1 mutations were more sensitive to PARP inhibitors [109].

The receptor tyrosine kinase c-Met has been found to bind and phosphorylate PARP1 at Tyr907 [110]. This is a resistance mechanism that does not restore the HR pathway, and phosphorylation of PARP1 can increase the enzyme activity of PARP1 and reduces binding to PARP inhibitors, resulting in insensitivity to PARP inhibitors [110]. c-Met is a proto-oncogene. In the case of BRCA inactivation, the expression of c-Met kinase is enhanced, so its expression in TNBC cell lines is higher than that in non-TNBC cell lines. Moreover, oxidative stress can induce nuclear transport of c-Met and its interaction with PARP1 [110]. TNBC cell growth was inhibited when c-Met inhibitor and PARP inhibitor were combined, further demonstrating this resistance mechanism.

In addition, a multicomponent analysis of breast tumors in genetically screened PARPi-sensitive and drug-resistant BRCA2 mutated mice determined that depletion of PAR glycohydrolase (PARG) restores PAR formation and partially rescues PARP1 signaling [111]. PARG depletion is more common in triple-negative breast cancer and serous ovarian cancer, leading treatment efficacy can not reach the expected.

## ALTERATIONS IN CELL CYCLE

**Figure 9:**
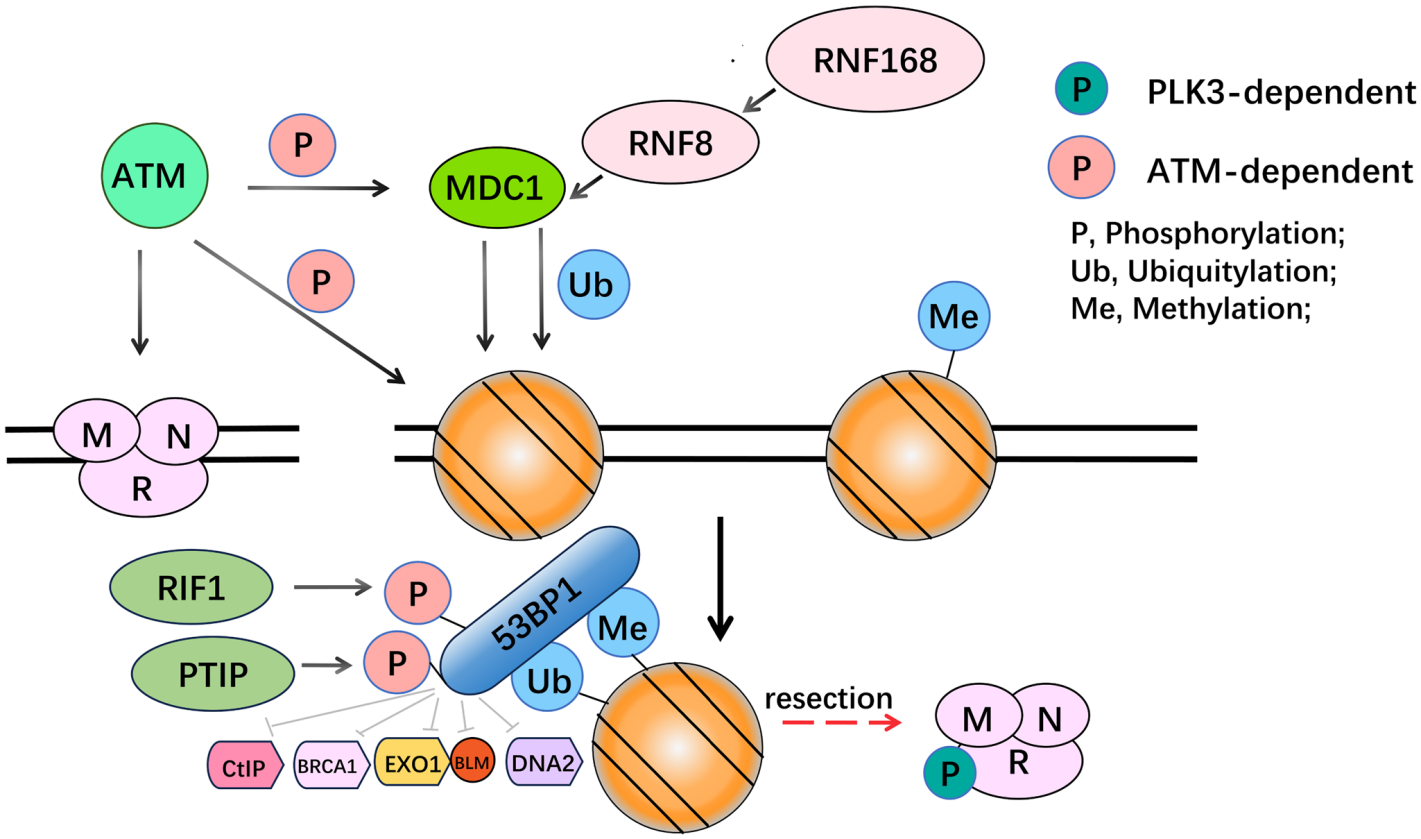
53BP1 intercepts DNA repair in cell cycle. By means of MRN, ATM is collected to DBS and phosphorylates targets, among which γH2AX recruits MDC1. MDC1 is also an ATM target. MDC1 phosphorylation gathers the E3 ubiquitin ligase RNF8, which can generate histone H2A ubiquitylation by recruiting another E3 ubiquitin ligase (RNF168). This modification with the addition of H4K20 methylation permits 53BP1 gathering. 53BP1 which is phosphorylated in an ATM-dependent way creates docking sites for RIF1 and PTIP therefore it blocks resection at DSBs.

DNA repair is regulated by the cell cycle [112], among which the most typical example is without doubt the periodic inhibition of DNA double-strand break (DBS) repair. In G1 and the early stage of S, the repair of double-strand breaks is influenced by some proteins, like 53BP1 and R1F1. Trough MRN, ATM is gathered into DSBs and targets for example nucleosomes (especially H2AX, resulting in γH2AX which can recruit MDC1) are phosphorylated. MDC1 phosphorylation contributes to histone H2A ubiquitylation by recruiting the E3 ubiquitin ligase (RNF8 and RNF168). Along with H4K20 methylation, this modification enables 53BP1 to recruit [113]. 53BP1 creates abutting joints for RIF1 and PTIP by phosphorylating in an ATM-dependent approach [114] (Figure 9). 53BP1 intercepts excision at DSBs, consequently leading DSB repair in the direction of NHEJ. Therefore, 53BP1 plays a vital role in the PARPi resistance [115].

Cyclin-dependent kinases (CDKs) activity is closely related to DNA end resection. Dinaciclib is an inhibitor of CDKs 1,2,5 and 9 that we all have known [116]. In models of triple-negative breast cancer (TNBC) which is an invasive breast carcinoma subgroup, dinaciclib as a CDK12 inhibitor cuts down the expression of HR gene in BCRA wild-type TNBC cells. And at the same time, dinaciclib improves the sensitivity of these cells to PARP inhibition. In addition, in triple-negative breast cancer (TNBC), on account of the deletion of CDK12, it can changeover both *De nova* PARPi resistance and acquired PARPi resistance, regardless of in BRCA wild-type or mutated models [117]. The expression of DNA replication genes is adjusted by CDK12, for example CDC6, CCNE1, and CDT1. Cyclin E1, the protein product of CCNE1, which can stimulate the progression of S phase, is overexpressed in high proliferation cancer cells. Cyclin E1 is phosphorylated by CDK12 at Ser366 position and this brings about the upregulation of cyclin E1 in cancer cells, for instance in the high-grade serous ovarian cancer (HGSC) [118]. Breast Cancer (BC) cells exists flaw in DNA double-strand break repair and, therefore, it is highly sensitive to PARP inhibition. While in BRCA1-mediated activating S phase checkpoint and cell multiplication, CDK-1 is necessity and overexpressed in BC cells [119]. Based on this point, in BC treatment resisting CDK-1 and PARP-1 jointly contributes to decreasing cell proliferation observably [120]. All of these evidences indicate that CDKs played a role in interdicting DNA end resistance and caused PARP inhibitor resistance. Combining PARPi and CDKs inhibitors and putting into therapy may make a difference promisingly [115].

## PHARMACOLOGICAL ALTERATION

Generalized pharmacological changes may also modulate PARP inhibitors resistance. Most of the approved PARP inhibitors currently on the market are substrates for the multidrug resistance protein (MDR1) encoded by ABCB1 [121], and the overexpression of MDR1 was associated with resistance to Olaparib.

When studying a new chemotherapy drug, it is necessary to confirm whether they are substrates for classical ABC transporters, such as P-glycoprotein (P-gp). Unfortunately, Olaparib seems to be a P-gp substrate, and P-gp overexpression models were found to be resistant to Olaparib in specific experimental studies [122]. Long-term treatment with Olaparib leads to upregulation of the Abcb1a/b gene encoding the P-gp efflux pump, which develops resistance, but this resistance can be reversed with the use of P-gp inhibitors [123].

Up to now, studies on the effect of pharmacological changes on PARP inhibitors resistance in clinical practice have not yet formed a system, and the underlying mechanism is still unclear.

## TUMOR MICROENVIRONMENT

TME plays a significant role in the initiation and promotion of TNBC, including promoting proliferation signaling, generating blood vessels, inhibiting cell apoptosis and evading immune surveillance [124]. TME is complex and heterogeneous, and heterogeneity is often considered a major challenge in breast cancer treatment [125]. In the early stages of tumor growth, tumor cells recruit relevant immune cells and matrix components to form a microenvironment that inhibits tumor inflammation, which is called TME. The interaction between cellular and non-cellular components in TME contributes greatly to the growth, invasion, metastasis and drug resistance of tumor cells [126].

TME is a very complex system composed of various types of cells and their secreted products (such as cytokines, chemokines) and other non-cellular components of the extracellular matrix.

Tumor-associated fibroblast CAF is a key ingredient in the breast cancer microenvironment, and it has been shown that the abundance of fibroblasts (CAF) in breast cancer promotes resistance to chemotherapy drugs [127].every subtype of CAF contributes to tumor growth promotion. Among them, the CAF-S1 subgroup of breast cancer promotes the immunosuppressive environment through a multistep mechanism and enhances the ability of regulatory T cells to inhibit the proliferation of T effectors [128]. In addition, fibroblasts will lack transforming growth factor-β-receptor-β-2 (TGFBR2) in the presence of chemotherapy drugs for the benefit of breast cancer cell survival and metastasis, but normal fibroblasts cannot achieve this process [129]. CAFs also generate the matrix metalloproteinases (MMPs) to promote cancer cell proliferation and recombination ECM [130].

It is essential for Tumor-associated macrophages (TAMs) in the process of drug resistance. As everyone knows, angiogenesis is a major mechanism of tumor recurrence. The CCL18 of Breast cancer TAM promotes the reconstitution of blood vessels around the tumor after chemotherapy [131]. In treatment with PARP inhibitors, although PARP inhibitors have toxic effects on HR deficient cancer cells, it has no toxic effects on HR intact cells in TME, such as TAM. PARP inhibitors can cause TAM to reprogram, causing its cytotoxicity and phagocytosis to become stronger [132]. In addition, PARP inhibitors also enhance the signal transduction and activator of transcription (STAT3) pathway in tumor cells, promoting the pro-tumor polarization of TAM [133].

Hypoxia is a typical feature of tumor microenvironment and an important intermediary of drug resistance. Hypoxia activates signal transduction of hypoxia-inducing molecules (HIF), which promote tumor growth and macrophage polarization [134]. Moreover, hypoxia also promotes the secretion of angiogenic factors, and the formation of blood vessels will positively promote the production of tumor cells [135].

The role of growth factors and chemokines in the regulation of TME should not be ignored. IL6, CSF2, CCL5 and VEGF are typical factors that regulate breast cancer traits [136]. Cancer-associated fibroblasts (CAMs) promote the cancer stem cell (CSC) phenotype in cancer cells by secreting CCL2 [137]. The CSC phenotype plays an important role in the development and drug resistance of cancer.

**Figure 10:**
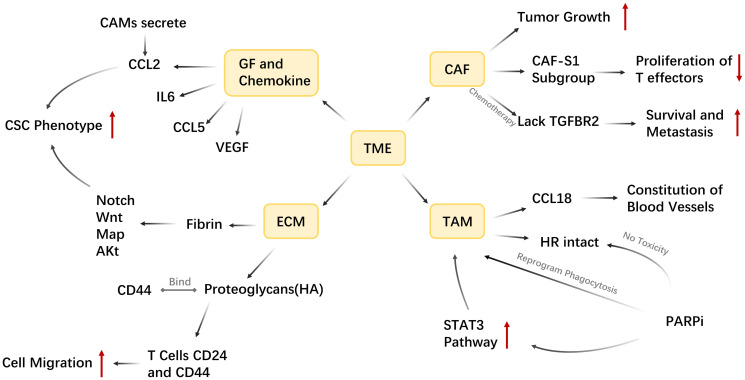
TME is a system composed four parts: GF and Chemokine, ECM, CAF and TAM. IL6, CCL5, VEG are typical growth factors in TME that regulate breast trait. CCL2 secreted by CAMS can promote CSC, and Fibrin in ECM also can achieve this by regulating Notch, Wnt, Map, Akt in cancer cells. When HA in ECM binds to CD44, the inhibitory activity of T cells CD24, CD25 decreases and promote cell migration. Moreover, the CAF-S1 subgroup of breast cancer enhances the ability of regulatory T cells and inhibits the proliferation of T effectors. In the presence of chemotherapy, fibroblast will lack TGFBR2 for the benefit of cancer cells survival and metastasis. The CCL8 in TAM can promote the constitution of blood vessels, which is a mechanism of tumor recurrence. When treating with PARPi, PARPi can cause TAM to reprogram to increase its cytotoxicity and enhance the STAT3 pathway to promote the pro-tumor polarization. And PARPi has no toxicity on HR intact cells in TAM.

Finally, let us understand more the non-cellular components of the extracellular matrix (ECM) of TME. ECM is an important complex scaffold of cancer cells, and its composition and content changes reflect the biological and physical characteristics to determine the fate of tumor cells. The main components of ECM are fibrin (such as collagen, elastin, fibronectin and laminin) and proteoglycans (such as chondroitin sulfate, heparan sulfate, keratin sulfate and hyaluronic acid) [130]. The composition of ECM determines the sensitivity of primary breast tumors to chemotherapy drugs. Fibrin regulates Notch, Wnt, MaP and Akt in cancer cells to promote CSC phenotype and provide support for cancer cell viability [127]. Hyaluronic acid (HA) in ECM binds to CD44, regulates the inhibitory activity of T cells CD24 and CD25, and plays a key role in cell migration and invasion [138]. ECM is increasingly recognized as an important regulator of breast cancer, and there is increasing evidence that ECM proteins induced in breast cancer play an important role in breast cancer progression and metastasis [139] (Figure 10).

In another way, PARP inhibitors induce DNA damage within tumor cells, leading to cell death and the release of DNA fragments. These fragments are recognized by the intracellular sensor cGAS, which activates the STING pathway, subsequently producing the second messenger cGAMP and triggering a series of immune responses. This process includes the activation of transcription factors IRF3 and NF-κB, promoting the expression of interferons and inflammatory cytokines, which in turn further enhance immune cell activation and antigen presentation. PARP inhibitors also impact the TME through upregulating PD-L1 expression on tumor cells and activating tumor-associated fibroblasts, which could contribute to tumor growth and dissemination [140].

## CONCLUSION

In summary, the mechanisms behind acquired and *de novo* resistance to PARP inhibitors are complex and multifaceted. A frequent route of acquired resistance to PARP inhibitors is the restoration of HR repair activity via BRCA1/2 reverse mutation, BRCA1 promoter methylation, and DNA end resection. Furthermore, PARP mutations or deletions, replication fork stabilization, suppression of DNA replication gaps, DSB repair pathway changes, epigenetic modifications, pharmacological alterations, and the tumor microenvironment all play significant functions in mediating resistance to PARP inhibitors. Understanding these mechanisms is essential for developing targeted therapeutic strategies to overcome PARPi resistance and improve patient outcomes in the treatment of ovarian cancer and other BRCA-mutated advanced cancers. Inhibiting the HR pathway is a prevalent strategy, wherein researchers are actively searching for small molecules that can impair the functions of BRCA1/2, as well as other crucial proteins in the HRR pathway, including CtIP, MRN complex, 53BP1, and POLQ, among others. Proteins belonging to the SNF2 family and MLL3/4 have been found to enhance the stability of replication forks and reduce stress-induced DNA breaks in cells lacking BRCA function. This knowledge could be leveraged for therapeutic development. Furthermore, the combination of PARG inhibitors with PARPi shows promise in clinical settings, as it increases patients’ sensitivity to PARPi by decreasing PARylation. Additionally, we have also found that improving the tumor microenvironment may also serve as a potential therapeutic approach to increase patients’ sensitivity to PARPi. Further clinical studies are both needed to fully elucidate these mechanisms and develop effective strategies to combat PARPi resistance.

## References

[1] Jackson SP, Bartek J. The DNA-damage response in human biology and disease. Nature. 2009; 461:1071–78. 10.1038/nature08467. 19847258 PMC2906700

[2] Carvalho JF, Kanaar R. Targeting homologous recombination-mediated DNA repair in cancer. Expert Opin Ther Targets. 2014; 18:427–58. 10.1517/14728222.2014.882900. 24491188

[3] Cortesi L, Rugo HS, Jackisch C. An Overview of PARP Inhibitors for the Treatment of Breast Cancer. Target Oncol. 2021; 16:255–82. 10.1007/s11523-021-00796-4. 33710534 PMC8105250

[4] Moynahan ME, Jasin M. Mitotic homologous recombination maintains genomic stability and suppresses tumorigenesis. Nat Rev Mol Cell Biol. 2010; 11:196–207. 10.1038/nrm2851. 20177395 PMC3261768

[5] Ashworth A, Lord CJ, Reis-Filho JS. Genetic interactions in cancer progression and treatment. Cell. 2011; 145:30–38. 10.1016/j.cell.2011.03.020. 21458666

[6] Langelier MF, Riccio AA, Pascal JM. PARP-2 and PARP-3 are selectively activated by 5’ phosphorylated DNA breaks through an allosteric regulatory mechanism shared with PARP-1. Nucleic Acids Res. 2014; 42:7762–75. 10.1093/nar/gku474. 24928857 PMC4081085

[7] Liu L, Kong M, Gassman NR, Freudenthal BD, Prasad R, Zhen S, Watkins SC, Wilson SH, Van Houten B. PARP1 changes from three-dimensional DNA damage searching to one-dimensional diffusion after auto-PARylation or in the presence of APE1. Nucleic Acids Res. 2017; 45:12834–47. 10.1093/nar/gkx1047. 29121337 PMC5728402

[8] Purnell MR, Whish WJ. Novel inhibitors of poly(ADP-ribose) synthetase. Biochem J. 1980; 185:775–77. 10.1042/bj1850775. 6248035 PMC1161458

[9] Davar D, Beumer JH, Hamieh L, Tawbi H. Role of PARP inhibitors in cancer biology and therapy. Curr Med Chem. 2012; 19:3907–21. 10.2174/092986712802002464. 22788767 PMC3421454

[10] Ferraris DV. Evolution of poly(ADP-ribose) polymerase-1 (PARP-1) inhibitors. From concept to clinic. J Med Chem. 2010; 53:4561–84. 10.1021/jm100012m. 20364863

[11] Trucco C, Oliver FJ, de Murcia G, Ménissier-de Murcia J. DNA repair defect in poly(ADP-ribose) polymerase-deficient cell lines. Nucleic Acids Res. 1998; 26:2644–49. 10.1093/nar/26.11.2644. 9592149 PMC147627

[12] Haber JE. DNA recombination: the replication connection. Trends Biochem Sci. 1999; 24:271–75. 10.1016/s0968-0004(99)01413-9. 10390616

[13] Karran P. DNA double strand break repair in mammalian cells. Curr Opin Genet Dev. 2000; 10:144–50. 10.1016/s0959-437x(00)00069-1. 10753787

[14] Arnaudeau C, Lundin C, Helleday T. DNA double-strand breaks associated with replication forks are predominantly repaired by homologous recombination involving an exchange mechanism in mammalian cells. J Mol Biol. 2001; 307:1235–45. 10.1006/jmbi.2001.4564. 11292338

[15] Gregg AV, McGlynn P, Jaktaji RP, Lloyd RG. Direct rescue of stalled DNA replication forks via the combined action of PriA and RecG helicase activities. Mol Cell. 2002; 9:241–51. 10.1016/s1097-2765(02)00455-0. 11864599

[16] Lomonosov M, Anand S, Sangrithi M, Davies R, Venkitaraman AR. Stabilization of stalled DNA replication forks by the BRCA2 breast cancer susceptibility protein. Genes Dev. 2003; 17:3017–22. 10.1101/gad.279003. 14681210 PMC305253

[17] Moynahan ME, Pierce AJ, Jasin M. BRCA2 is required for homology-directed repair of chromosomal breaks. Mol Cell. 2001; 7:263–72. 10.1016/s1097-2765(01)00174-5. 11239455

[18] Amé JC, Rolli V, Schreiber V, Niedergang C, Apiou F, Decker P, Muller S, Höger T, Ménissier-de Murcia J, de Murcia G. PARP-2, A novel mammalian DNA damage-dependent poly(ADP-ribose) polymerase. J Biol Chem. 1999; 274:17860–68. 10.1074/jbc.274.25.17860. 10364231

[19] Heyer BS, MacAuley A, Behrendtsen O, Werb Z. Hypersensitivity to DNA damage leads to increased apoptosis during early mouse development. Genes Dev. 2000; 14:2072–84. 10950870 PMC316856

[20] Schreiber V, Amé JC, Dollé P, Schultz I, Rinaldi B, Fraulob V, Ménissier-de Murcia J, de Murcia G. Poly(ADP-ribose) polymerase-2 (PARP-2) is required for efficient base excision DNA repair in association with PARP-1 and XRCC1. J Biol Chem. 2002; 277:23028–36. 10.1074/jbc.M202390200. 11948190

[21] Zhang J, Stevens MF, Bradshaw TD. Temozolomide: mechanisms of action, repair and resistance. Curr Mol Pharmacol. 2012; 5:102–14. 10.2174/1874467211205010102. 22122467

[22] Kutuzov MM, Khodyreva SN, Amé JC, Ilina ES, Sukhanova MV, Schreiber V, Lavrik OI. Interaction of PARP-2 with DNA structures mimicking DNA repair intermediates and consequences on activity of base excision repair proteins. Biochimie. 2013; 95:1208–15. 10.1016/j.biochi.2013.01.007. 23357680

[23] Murata MM, Kong X, Moncada E, Chen Y, Imamura H, Wang P, Berns MW, Yokomori K, Digman MA. NAD+ consumption by PARP1 in response to DNA damage triggers metabolic shift critical for damaged cell survival. Mol Biol Cell. 2019; 30:2584–97. 10.1091/mbc.E18-10-0650. 31390283 PMC6740200

[24] Pleschke JM, Kleczkowska HE, Strohm M, Althaus FR. Poly(ADP-ribose) binds to specific domains in DNA damage checkpoint proteins. J Biol Chem. 2000; 275:40974–80. 10.1074/jbc.M006520200. 11016934

[25] Dantzer F, de La Rubia G, Ménissier-De Murcia J, Hostomsky Z, de Murcia G, Schreiber V. Base excision repair is impaired in mammalian cells lacking Poly(ADP-ribose) polymerase-1. Biochemistry. 2000; 39:7559–69. 10.1021/bi0003442. 10858306

[26] Qi H, Grace Wright RH, Beato M, Price BD. The ADP-ribose hydrolase NUDT5 is important for DNA repair. Cell Rep. 2022; 41:111866. 10.1016/j.celrep.2022.111866. 36543120

[27] Lockett KL, Snowhite IV, Hu JJ. Nucleotide-excision repair and prostate cancer risk. Cancer Lett. 2005; 220:125–35. 10.1016/j.canlet.2004.08.019. 15766587

[28] Pommier Y, O’Connor MJ, de Bono J. Laying a trap to kill cancer cells: PARP inhibitors and their mechanisms of action. Sci Transl Med. 2016; 8:362ps17. 10.1126/scitranslmed.aaf9246. 27797957

[29] Mortusewicz O, Amé JC, Schreiber V, Leonhardt H. Feedback-regulated poly(ADP-ribosyl)ation by PARP-1 is required for rapid response to DNA damage in living cells. Nucleic Acids Res. 2007; 35:7665–75. 10.1093/nar/gkm933. 17982172 PMC2190722

[30] Godon C, Cordelières FP, Biard D, Giocanti N, Mégnin-Chanet F, Hall J, Favaudon V. PARP inhibition versus PARP-1 silencing: different outcomes in terms of single-strand break repair and radiation susceptibility. Nucleic Acids Res. 2008; 36:4454–64. 10.1093/nar/gkn403. 18603595 PMC2490739

[31] Okano S, Lan L, Caldecott KW, Mori T, Yasui A. Spatial and temporal cellular responses to single-strand breaks in human cells. Mol Cell Biol. 2003; 23:3974–81. 10.1128/MCB.23.11.3974-3981.2003. 12748298 PMC155230

[32] Murai J, Huang SY, Das BB, Renaud A, Zhang Y, Doroshow JH, Ji J, Takeda S, Pommier Y. Trapping of PARP1 and PARP2 by Clinical PARP Inhibitors. Cancer Res. 2012; 72:5588–99. 10.1158/0008-5472.CAN-12-2753. 23118055 PMC3528345

[33] Murai J, Zhang Y, Morris J, Ji J, Takeda S, Doroshow JH, Pommier Y. Rationale for poly(ADP-ribose) polymerase (PARP) inhibitors in combination therapy with camptothecins or temozolomide based on PARP trapping versus catalytic inhibition. J Pharmacol Exp Ther. 2014; 349:408–16. 10.1124/jpet.113.210146. 24650937 PMC4019318

[34] Ström CE, Johansson F, Uhlén M, Szigyarto CA, Erixon K, Helleday T. Poly (ADP-ribose) polymerase (PARP) is not involved in base excision repair but PARP inhibition traps a single-strand intermediate. Nucleic Acids Res. 2011; 39:3166–75. 10.1093/nar/gkq1241. 21183466 PMC3082910

[35] Hopkins TA, Shi Y, Rodriguez LE, Solomon LR, Donawho CK, DiGiammarino EL, Panchal SC, Wilsbacher JL, Gao W, Olson AM, Stolarik DF, Osterling DJ, Johnson EF, Maag D. Mechanistic Dissection of PARP1 Trapping and the Impact on In Vivo Tolerability and Efficacy of PARP Inhibitors. Mol Cancer Res. 2015; 13:1465–77. 10.1158/1541-7786.MCR-15-0191-T. 26217019

[36] Weigelt B, Comino-Méndez I, de Bruijn I, Tian L, Meisel JL, García-Murillas I, Fribbens C, Cutts R, Martelotto LG, Ng CKY, Lim RS, Selenica P, Piscuoglio S, et al. Diverse *BRCA1* and *BRCA2* Reversion Mutations in Circulating Cell-Free DNA of Therapy-Resistant Breast or Ovarian Cancer. Clin Cancer Res. 2017; 23:6708–20. 10.1158/1078-0432.CCR-17-0544. 28765325 PMC5728372

[37] Dev H, Chiang TW, Lescale C, de Krijger I, Martin AG, Pilger D, Coates J, Sczaniecka-Clift M, Wei W, Ostermaier M, Herzog M, Lam J, Shea A, et al. Shieldin complex promotes DNA end-joining and counters homologous recombination in BRCA1-null cells. Nat Cell Biol. 2018; 20:954–65. 10.1038/s41556-018-0140-1. 30022119 PMC6145444

[38] D’Andrea AD. Mechanisms of PARP inhibitor sensitivity and resistance. DNA Repair (Amst). 2018; 71:172–76. 10.1016/j.dnarep.2018.08.021. 30177437

[39] Barber LJ, Sandhu S, Chen L, Campbell J, Kozarewa I, Fenwick K, Assiotis I, Rodrigues DN, Reis Filho JS, Moreno V, Mateo J, Molife LR, De Bono J, et al. Secondary mutations in BRCA2 associated with clinical resistance to a PARP inhibitor. J Pathol. 2013; 229:422–29. 10.1002/path.4140. 23165508

[40] Christie EL, Fereday S, Doig K, Pattnaik S, Dawson SJ, Bowtell DDL. Reversion of BRCA1/2 Germline Mutations Detected in Circulating Tumor DNA From Patients With High-Grade Serous Ovarian Cancer. J Clin Oncol. 2017; 35:1274–80. 10.1200/JCO.2016.70.4627. 28414925

[41] Drost R, Bouwman P, Rottenberg S, Boon U, Schut E, Klarenbeek S, Klijn C, van der Heijden I, van der Gulden H, Wientjens E, Pieterse M, Catteau A, Green P, et al. BRCA1 RING function is essential for tumor suppression but dispensable for therapy resistance. Cancer Cell. 2011; 20:797–809. 10.1016/j.ccr.2011.11.014. 22172724

[42] Edwards SL, Brough R, Lord CJ, Natrajan R, Vatcheva R, Levine DA, Boyd J, Reis-Filho JS, Ashworth A. Resistance to therapy caused by intragenic deletion in BRCA2. Nature. 2008; 451:1111–15. 10.1038/nature06548. 18264088

[43] Carneiro BA, Collier KA, Nagy RJ, Pamarthy S, Sagar V, Fairclough S, Odegaard J, Lanman RB, Costa R, Taxter T, Kuzel TM, Fan A, Chae YK, et al. Acquired Resistance to Poly (ADP-ribose) Polymerase Inhibitor Olaparib in *BRCA2*-Associated Prostate Cancer Resulting From Biallelic *BRCA2* Reversion Mutations Restores Both Germline and Somatic Loss-of-Function Mutations. JCO Precis Oncol. 2018; 2:PO.17.00176. 10.1200/PO.17.00176. 31501807 PMC6732782

[44] Lin KK, Harrell MI, Oza AM, Oaknin A, Ray-Coquard I, Tinker AV, Helman E, Radke MR, Say C, Vo LT, Mann E, Isaacson JD, Maloney L, et al. *BRCA* Reversion Mutations in Circulating Tumor DNA Predict Primary and Acquired Resistance to the PARP Inhibitor Rucaparib in High-Grade Ovarian Carcinoma. Cancer Discov. 2019; 9:210–19. 10.1158/2159-8290.CD-18-0715. 30425037

[45] Kondrashova O, Topp M, Nesic K, Lieschke E, Ho GY, Harrell MI, Zapparoli GV, Hadley A, Holian R, Boehm E, Heong V, Sanij E, Pearson RB, et al, and Australian Ovarian Cancer Study (AOCS). Methylation of all BRCA1 copies predicts response to the PARP inhibitor rucaparib in ovarian carcinoma. Nat Commun. 2018; 9:3970. 10.1038/s41467-018-05564-z. 30266954 PMC6162272

[46] Bhin J, Paes Dias M, Gogola E, Rolfs F, Piersma SR, de Bruijn R, de Ruiter JR, van den Broek B, Duarte AA, Sol W, van der Heijden I, Andronikou C, Kaiponen TS, et al. Multi-omics analysis reveals distinct non-reversion mechanisms of PARPi resistance in BRCA1- versus BRCA2-deficient mammary tumors. Cell Rep. 2023; 42:112538. 10.1016/j.celrep.2023.112538. 37209095 PMC10242444

[47] Xu Y, Xu D. Repair pathway choice for double-strand breaks. Essays Biochem. 2020; 64:765–77. 10.1042/EBC20200007. 32648897

[48] Seol JH, Shim EY, Lee SE. Microhomology-mediated end joining: Good, bad and ugly. Mutat Res. 2018; 809:81–87. 10.1016/j.mrfmmm.2017.07.002. 28754468 PMC6477918

[49] Zhou Q, Tu X, Hou X, Yu J, Zhao F, Huang J, Kloeber J, Olson A, Gao M, Luo K, Zhu S, Wu Z, Zhang Y, et al. Syk-dependent alternative homologous recombination activation promotes cancer resistance to DNA targeted therapy. Res Sq [Preprint]. 2023; rs.3.rs-2922520. 10.21203/rs.3.rs-2922520/v1. . Update in: Drug Resist Updat. 2024; 74:101085. 10.21203/rs.3.rs-2922520/v1. 38636338 PMC11095636

[50] Koppensteiner R, Samartzis EP, Noske A, von Teichman A, Dedes I, Gwerder M, Imesch P, Ikenberg K, Moch H, Fink D, Stucki M, Dedes KJ. Effect of MRE11 loss on PARP-inhibitor sensitivity in endometrial cancer in vitro. PLoS One. 2014; 9:e100041. 10.1371/journal.pone.0100041. 24927325 PMC4057395

[51] Brandt S, Samartzis EP, Zimmermann AK, Fink D, Moch H, Noske A, Dedes KJ. Lack of MRE11-RAD50-NBS1 (MRN) complex detection occurs frequently in low-grade epithelial ovarian cancer. BMC Cancer. 2017; 17:44. 10.1186/s12885-016-3026-2. 28073364 PMC5223425

[52] Kim JJ, Lee SY, Choi JH, Woo HG, Xhemalce B, Miller KM. PCAF-Mediated Histone Acetylation Promotes Replication Fork Degradation by MRE11 and EXO1 in BRCA-Deficient Cells. Mol Cell. 2020; 80:327–44.e8. 10.1016/j.molcel.2020.08.018. 32966758 PMC7572766

[53] Gupta R, Somyajit K, Narita T, Maskey E, Stanlie A, Kremer M, Typas D, Lammers M, Mailand N, Nussenzweig A, Lukas J, Choudhary C. DNA Repair Network Analysis Reveals Shieldin as a Key Regulator of NHEJ and PARP Inhibitor Sensitivity. Cell. 2018; 173:972–88.e23. 10.1016/j.cell.2018.03.050. 29656893 PMC8108093

[54] Mirman Z, de Lange T. 53BP1: a DSB escort. Genes Dev. 2020; 34:7–23. 10.1101/gad.333237.119. 31896689 PMC6938671

[55] Clairmont CS, D’Andrea AD. REV7 directs DNA repair pathway choice. Trends Cell Biol. 2021; 31:965–78. 10.1016/j.tcb.2021.05.009. 34147298 PMC8608404

[56] Murakumo Y, Sakurai Y, Kato T, Hashimoto H, Ichinoe M. REV7 in Cancer Biology and Management. Cancers (Basel). 2023; 15:1721. 10.3390/cancers15061721. 36980607 PMC10046837

[57] Jiang X, Li X, Li W, Bai H, Zhang Z. PARP inhibitors in ovarian cancer: Sensitivity prediction and resistance mechanisms. J Cell Mol Med. 2019; 23:2303–13. 10.1111/jcmm.14133. 30672100 PMC6433712

[58] Ceccaldi R, Liu JC, Amunugama R, Hajdu I, Primack B, Petalcorin MI, O’Connor KW, Konstantinopoulos PA, Elledge SJ, Boulton SJ, Yusufzai T, D’Andrea AD. Homologous-recombination-deficient tumours are dependent on Polθ-mediated repair. Nature. 2015; 518:258–62. 10.1038/nature14184. 25642963 PMC4415602

[59] Belan O, Sebald M, Adamowicz M, Anand R, Vancevska A, Neves J, Grinkevich V, Hewitt G, Segura-Bayona S, Bellelli R, Robinson HMR, Higgins GS, Smith GCM, et al. POLQ seals post-replicative ssDNA gaps to maintain genome stability in BRCA-deficient cancer cells. Mol Cell. 2022; 82:4664–80.e9. 10.1016/j.molcel.2022.11.008. 36455556

[60] Tomimatsu N, Mukherjee B, Catherine Hardebeck M, Ilcheva M, Vanessa Camacho C, Louise Harris J, Porteus M, Llorente B, Khanna KK, Burma S. Phosphorylation of EXO1 by CDKs 1 and 2 regulates DNA end resection and repair pathway choice. Nat Commun. 2014; 5:3561. 10.1038/ncomms4561. 24705021 PMC4041212

[61] Ning JF, Stanciu M, Humphrey MR, Gorham J, Wakimoto H, Nishihara R, Lees J, Zou L, Martuza RL, Wakimoto H, Rabkin SD. Myc targeted CDK18 promotes ATR and homologous recombination to mediate PARP inhibitor resistance in glioblastoma. Nat Commun. 2019; 10:2910. 10.1038/s41467-019-10993-5. 31266951 PMC6606647

[62] Militello AM, Zielli T, Boggiani D, Michiara M, Naldi N, Bortesi B, Zanelli P, Uliana V, Giuliotti S, Musolino A. Mechanism of Action and Clinical Efficacy of CDK4/6 Inhibitors in BRCA-Mutated, Estrogen Receptor-Positive Breast Cancers: Case Report and Literature Review. Front Oncol. 2019; 9:759. 10.3389/fonc.2019.00759. 31456944 PMC6700293

[63] Quereda V, Bayle S, Vena F, Frydman SM, Monastyrskyi A, Roush WR, Duckett DR. Therapeutic Targeting of CDK12/CDK13 in Triple-Negative Breast Cancer. Cancer Cell. 2019; 36:545–58.e7. 10.1016/j.ccell.2019.09.004. 31668947

[64] Bakr A, Hey J, Sigismondo G, Liu CS, Sadik A, Goyal A, Cross A, Iyer RL, Müller P, Trauernicht M, Breuer K, Lutsik P, Opitz CA, et al. ID3 promotes homologous recombination via non-transcriptional and transcriptional mechanisms and its loss confers sensitivity to PARP inhibition. Nucleic Acids Res. 2021; 49:11666–89. 10.1093/nar/gkab964. 34718742 PMC8599806

[65] Hu K, Li Y, Wu W, Xie L, Yan H, Cai Y, Chen D, Jiang Q, Lin L, Chen Z, Liao JY, Zhang Y, Koeffler HP, et al. ATM-Dependent Recruitment of BRD7 is required for Transcriptional Repression and DNA Repair at DNA Breaks Flanking Transcriptional Active Regions. Adv Sci (Weinh). 2020; 7:2000157. 10.1002/advs.202000157. 33101843 PMC7578904

[66] Sun Y, Ning X, Fan J, Hu J, Jiang Y, Hu Z, Paulo JA, Liu J, Qiu X, Xu H, Fu S, Gygi SP, Zhang J, Zhou C. Loss of tumor suppressor inositol polyphosphate 4-phosphatase type B impairs DNA double-strand break repair by destabilization of DNA tethering protein Rad50. Cell Death Dis. 2020; 11:292. 10.1038/s41419-020-2491-3. 32341333 PMC7184567

[67] Merentitis D, Nguyen BD, Samartzis EP, Noske A, Brandt S, Dedes KJ. Loss of MDC1 in Endometrial Carcinoma Is Associated With Loss of MRN Complex and MMR Deficiency. Anticancer Res. 2019; 39:6547–53. 10.21873/anticanres.13870. 31810920

[68] Li Z, Li J, Kong Y, Yan S, Ahmad N, Liu X. Plk1 Phosphorylation of Mre11 Antagonizes the DNA Damage Response. Cancer Res. 2017; 77:3169–80. 10.1158/0008-5472.CAN-16-2787. 28512243 PMC5504882

[69] O’Sullivan J, Kothari C, Caron MC, Gagné JP, Jin Z, Nonfoux L, Beneyton A, Coulombe Y, Thomas M, Atalay N, Meng XW, Milano L, Jean D, et al. ZNF432 stimulates PARylation and inhibits DNA resection to balance PARPi sensitivity and resistance. Nucleic Acids Res. 2023; 51:11056–79. 10.1093/nar/gkad791. 37823600 PMC10639050

[70] Lu Z, Mao W, Yang H, Santiago-O’Farrill JM, Rask PJ, Mondal J, Chen H, Ivan C, Liu X, Liu CG, Xi Y, Masuda K, Carrami EM, et al. SIK2 inhibition enhances PARP inhibitor activity synergistically in ovarian and triple-negative breast cancers. J Clin Invest. 2022; 132:e146471. 10.1172/JCI146471. 35642638 PMC9151707

[71] Tkáč J, Xu G, Adhikary H, Young JTF, Gallo D, Escribano-Díaz C, Krietsch J, Orthwein A, Munro M, Sol W, Al-Hakim A, Lin ZY, Jonkers J, et al. HELB Is a Feedback Inhibitor of DNA End Resection. Mol Cell. 2016; 61:405–18. 10.1016/j.molcel.2015.12.013. 26774285

[72] Eustermann S, Wu WF, Langelier MF, Yang JC, Easton LE, Riccio AA, Pascal JM, Neuhaus D. Structural Basis of Detection and Signaling of DNA Single-Strand Breaks by Human PARP-1. Mol Cell. 2015; 60:742–54. 10.1016/j.molcel.2015.10.032. 26626479 PMC4678113

[73] Slade D. PARP and PARG inhibitors in cancer treatment. Genes Dev. 2020; 34:360–94. 10.1101/gad.334516.119. 32029455 PMC7050487

[74] Hanzlikova H, Gittens W, Krejcikova K, Zeng Z, Caldecott KW. Overlapping roles for PARP1 and PARP2 in the recruitment of endogenous XRCC1 and PNKP into oxidized chromatin. Nucleic Acids Res. 2017; 45:2546–57. 10.1093/nar/gkw1246. 27965414 PMC5389470

[75] McLornan DP, List A, Mufti GJ. Applying synthetic lethality for the selective targeting of cancer. N Engl J Med. 2014; 371:1725–35. 10.1056/NEJMra1407390. 25354106

[76] Alemasova EE, Lavrik OI. Poly(ADP-ribosyl)ation by PARP1: reaction mechanism and regulatory proteins. Nucleic Acids Res. 2019; 47:3811–27. 10.1093/nar/gkz120. 30799503 PMC6486540

[77] Dawicki-McKenna JM, Langelier MF, DeNizio JE, Riccio AA, Cao CD, Karch KR, McCauley M, Steffen JD, Black BE, Pascal JM. PARP-1 Activation Requires Local Unfolding of an Autoinhibitory Domain. Mol Cell. 2015; 60:755–68. 10.1016/j.molcel.2015.10.013. 26626480 PMC4712911

[78] Makvandi M, Pantel A, Schwartz L, Schubert E, Xu K, Hsieh CJ, Hou C, Kim H, Weng CC, Winters H, Doot R, Farwell MD, Pryma DA, et al. A PET imaging agent for evaluating PARP-1 expression in ovarian cancer. J Clin Invest. 2018; 128:2116–26. 10.1172/JCI97992. 29509546 PMC5919879

[79] Thomas A, Murai J, Pommier Y. The evolving landscape of predictive biomarkers of response to PARP inhibitors. J Clin Invest. 2018; 128:1727–30. 10.1172/JCI120388. 29664016 PMC5919798

[80] Pettitt SJ, Krastev DB, Brandsma I, Dréan A, Song F, Aleksandrov R, Harrell MI, Menon M, Brough R, Campbell J, Frankum J, Ranes M, Pemberton HN, et al. Genome-wide and high-density CRISPR-Cas9 screens identify point mutations in PARP1 causing PARP inhibitor resistance. Nat Commun. 2018; 9:1849. 10.1038/s41467-018-03917-2. 29748565 PMC5945626

[81] Santofimia-Castaño P, Huang C, Liu X, Xia Y, Audebert S, Camoin L, Peng L, Lomberk G, Urrutia R, Soubeyran P, Neira JL, Iovanna J. NUPR1 protects against hyperPARylation-dependent cell death. Commun Biol. 2022; 5:732. 10.1038/s42003-022-03705-1. 35869257 PMC9307593

[82] Zhu H, Wei M, Xu J, Hua J, Liang C, Meng Q, Zhang Y, Liu J, Zhang B, Yu X, Shi S. PARP inhibitors in pancreatic cancer: molecular mechanisms and clinical applications. Mol Cancer. 2020; 19:49. 10.1186/s12943-020-01167-9. 32122376 PMC7053129

[83] Ray Chaudhuri A, Callen E, Ding X, Gogola E, Duarte AA, Lee JE, Wong N, Lafarga V, Calvo JA, Panzarino NJ, John S, Day A, Crespo AV, et al. Replication fork stability confers chemoresistance in BRCA-deficient cells. Nature. 2016; 535:382–87. 10.1038/nature18325. 27443740 PMC4959813

[84] Berti M, Cortez D, Lopes M. The plasticity of DNA replication forks in response to clinically relevant genotoxic stress. Nat Rev Mol Cell Biol. 2020; 21:633–51. 10.1038/s41580-020-0257-5. 32612242

[85] Kondratick CM, Washington MT, Spies M. Making Choices: DNA Replication Fork Recovery Mechanisms. Semin Cell Dev Biol. 2021; 113:27–37. 10.1016/j.semcdb.2020.10.001. 33967572 PMC8098667

[86] Jackson LM, Moldovan GL. Mechanisms of PARP1 inhibitor resistance and their implications for cancer treatment. NAR Cancer. 2022; 4:zcac042. 10.1093/narcan/zcac042. 36568963 PMC9773381

[87] Taglialatela A, Alvarez S, Leuzzi G, Sannino V, Ranjha L, Huang JW, Madubata C, Anand R, Levy B, Rabadan R, Cejka P, Costanzo V, Ciccia A. Restoration of Replication Fork Stability in BRCA1- and BRCA2-Deficient Cells by Inactivation of SNF2-Family Fork Remodelers. Mol Cell. 2017; 68:414–30.e8. 10.1016/j.molcel.2017.09.036. 29053959 PMC5720682

[88] Hopfner KP. Mre11-Rad50: the DNA end game. Biochem Soc Trans. 2023; 51:527–38. 10.1042/BST20220754. 36892213

[89] Schlacher K, Christ N, Siaud N, Egashira A, Wu H, Jasin M. Double-strand break repair-independent role for BRCA2 in blocking stalled replication fork degradation by MRE11. Cell. 2011; 145:529–42. 10.1016/j.cell.2011.03.041. 21565612 PMC3261725

[90] Ning H, Huang S, Lei Y, Zhi R, Yan H, Jin J, Hu Z, Guo K, Liu J, Yang J, Liu Z, Ba Y, Gao X, Hu D. Enhancer decommissioning by MLL4 ablation elicits dsRNA-interferon signaling and GSDMD-mediated pyroptosis to potentiate anti-tumor immunity. Nat Commun. 2022; 13:6578. 10.1038/s41467-022-34253-1. 36323669 PMC9630274

[91] Wang Q, Hu J, Han G, Wang P, Li S, Chang J, Gao K, Yin R, Li Y, Zhang T, Chai J, Gao Z, Zhang T, et al. PTIP governs NAD^+^ metabolism by regulating CD38 expression to drive macrophage inflammation. Cell Rep. 2022; 38:110603. 10.1016/j.celrep.2022.110603. 35354042

[92] Roy S, Tomaszowski KH, Luzwick JW, Park S, Li J, Murphy M, Schlacher K. p53 orchestrates DNA replication restart homeostasis by suppressing mutagenic RAD52 and POLθ pathways. Elife. 2018; 7:e31723. 10.7554/eLife.31723. 29334356 PMC5832412

[93] Guillemette S, Serra RW, Peng M, Hayes JA, Konstantinopoulos PA, Green MR, Cantor SB. Resistance to therapy in BRCA2 mutant cells due to loss of the nucleosome remodeling factor CHD4. Genes Dev. 2015; 29:489–94. 10.1101/gad.256214.114. 25737278 PMC4358401

[94] Bryant HE, Petermann E, Schultz N, Jemth AS, Loseva O, Issaeva N, Johansson F, Fernandez S, McGlynn P, Helleday T. PARP is activated at stalled forks to mediate Mre11-dependent replication restart and recombination. EMBO J. 2009; 28:2601–15. 10.1038/emboj.2009.206. 19629035 PMC2738702

[95] Wang Y, Luo W, Wang Y. PARP-1 and its associated nucleases in DNA damage response. DNA Repair (Amst). 2019; 81:102651. 10.1016/j.dnarep.2019.102651. 31302005 PMC6764844

[96] Cong K, Peng M, Kousholt AN, Lee WTC, Lee S, Nayak S, Krais J, VanderVere-Carozza PS, Pawelczak KS, Calvo J, Panzarino NJ, Turchi JJ, Johnson N, et al. Replication gaps are a key determinant of PARP inhibitor synthetic lethality with BRCA deficiency. Mol Cell. 2021; 81:3128–44.e7. 10.1016/j.molcel.2021.06.011. 34216544 PMC9089372

[97] Hanzlikova H, Kalasova I, Demin AA, Pennicott LE, Cihlarova Z, Caldecott KW. The Importance of Poly(ADP-Ribose) Polymerase as a Sensor of Unligated Okazaki Fragments during DNA Replication. Mol Cell. 2018; 71:319–31.e3. 10.1016/j.molcel.2018.06.004. 29983321 PMC6060609

[98] Tirman S, Quinet A, Wood M, Meroni A, Cybulla E, Jackson J, Pegoraro S, Simoneau A, Zou L, Vindigni A. Temporally distinct post-replicative repair mechanisms fill PRIMPOL-dependent ssDNA gaps in human cells. Mol Cell. 2021; 81:4026–40.e8. 10.1016/j.molcel.2021.09.013. 34624216 PMC8555837

[99] Bellí G, Colomina N, Castells-Roca L, Lorite NP. Post-Translational Modifications of PCNA: Guiding for the Best DNA Damage Tolerance Choice. J Fungi (Basel). 2022; 8:621. 10.3390/jof8060621. 35736104 PMC9225081

[100] Drzewiecka M, Barszczewska-Pietraszek G, Czarny P, Skorski T, Śliwiński T. Synthetic Lethality Targeting Polθ. Genes (Basel). 2022; 13:1101. 10.3390/genes13061101. 35741863 PMC9223150

[101] Prodhomme MK, Péricart S, Pommier RM, Morel AP, Brunac AC, Franchet C, Moyret-Lalle C, Brousset P, Puisieux A, Hoffmann JS, Tissier A. Opposite Roles for ZEB1 and TMEJ in the Regulation of Breast Cancer Genome Stability. Front Cell Dev Biol. 2021; 9:727429. 10.3389/fcell.2021.727429. 34458275 PMC8388841

[102] Harahap WA, Sudji IR, Nindrea RD. BRCA1 Promoter Methylation and Clinicopathological Characteristics in Sporadic Breast Cancer Patients in Indonesia. Asian Pac J Cancer Prev. 2018; 19:2643–49. 10.22034/APJCP.2018.19.9.2643. 30256562 PMC6249447

[103] Poh W, Dilley RL, Moliterno AR, Maciejewski JP, Pratz KW, McDevitt MA, Herman JG. *BRCA1* Promoter Methylation Is Linked to Defective Homologous Recombination Repair and Elevated *miR-155* to Disrupt Myeloid Differentiation in Myeloid Malignancies. Clin Cancer Res. 2019; 25:2513–22. 10.1158/1078-0432.CCR-18-0179. 30692098

[104] Ter Brugge P, Kristel P, van der Burg E, Boon U, de Maaker M, Lips E, Mulder L, de Ruiter J, Moutinho C, Gevensleben H, Marangoni E, Majewski I, Józwiak K, et al. Mechanisms of Therapy Resistance in Patient-Derived Xenograft Models of BRCA1-Deficient Breast Cancer. J Natl Cancer Inst. 2016; 108. 10.1093/jnci/djw148. 27381626

[105] Lei T, Du S, Peng Z, Chen L. Multifaceted regulation and functions of 53BP1 in NHEJ-mediated DSB repair (Review). Int J Mol Med. 2022; 50:90. 10.3892/ijmm.2022.5145. 35583003 PMC9162042

[106] Guo X, Bai Y, Zhao M, Zhou M, Shen Q, Yun CH, Zhang H, Zhu WG, Wang J. Acetylation of 53BP1 dictates the DNA double strand break repair pathway. Nucleic Acids Res. 2018; 46:689–703. 10.1093/nar/gkx1208. 29190394 PMC5778472

[107] Bunting SF, Callén E, Wong N, Chen HT, Polato F, Gunn A, Bothmer A, Feldhahn N, Fernandez-Capetillo O, Cao L, Xu X, Deng CX, Finkel T, et al. 53BP1 inhibits homologous recombination in Brca1-deficient cells by blocking resection of DNA breaks. Cell. 2010; 141:243–54. 10.1016/j.cell.2010.03.012. 20362325 PMC2857570

[108] Oplustilova L, Wolanin K, Mistrik M, Korinkova G, Simkova D, Bouchal J, Lenobel R, Bartkova J, Lau A, O’Connor MJ, Lukas J, Bartek J. Evaluation of candidate biomarkers to predict cancer cell sensitivity or resistance to PARP-1 inhibitor treatment. Cell Cycle. 2012; 11:3837–50. 10.4161/cc.22026. 22983061 PMC3495826

[109] Pettitt SJ, Rehman FL, Bajrami I, Brough R, Wallberg F, Kozarewa I, Fenwick K, Assiotis I, Chen L, Campbell J, Lord CJ, Ashworth A. A genetic screen using the PiggyBac transposon in haploid cells identifies Parp1 as a mediator of olaparib toxicity. PLoS One. 2013; 8:e61520. 10.1371/journal.pone.0061520. 23634208 PMC3636235

[110] Du Y, Yamaguchi H, Wei Y, Hsu JL, Wang HL, Hsu YH, Lin WC, Yu WH, Leonard PG, Lee GR 4th, Chen MK, Nakai K, Hsu MC, et al. Blocking c-Met-mediated PARP1 phosphorylation enhances anti-tumor effects of PARP inhibitors. Nat Med. 2016; 22:194–201. 10.1038/nm.4032. 26779812 PMC4754671

[111] Gogola E, Duarte AA, de Ruiter JR, Wiegant WW, Schmid JA, de Bruijn R, James DI, Guerrero Llobet S, Vis DJ, Annunziato S, van den Broek B, Barazas M, Kersbergen A, et al. Selective Loss of PARG Restores PARylation and Counteracts PARP Inhibitor-Mediated Synthetic Lethality. Cancer Cell. 2018; 33:1078–93.e12. 10.1016/j.ccell.2018.05.008. 29894693

[112] Murray JM, Carr AM. Integrating DNA damage repair with the cell cycle. Curr Opin Cell Biol. 2018; 52:120–5. 10.1016/j.ceb.2018.03.006. 29587168

[113] Hustedt N, Durocher D. The control of DNA repair by the cell cycle. Nat Cell Biol. 2016; 19:1–9. 10.1038/ncb3452. 28008184

[114] Escribano-Díaz C, Orthwein A, Fradet-Turcotte A, Xing M, Young JT, Tkáč J, Cook MA, Rosebrock AP, Munro M, Canny MD, Xu D, Durocher D. A cell cycle-dependent regulatory circuit composed of 53BP1-RIF1 and BRCA1-CtIP controls DNA repair pathway choice. Mol Cell. 2013; 49:872–83. 10.1016/j.molcel.2013.01.001. 23333306

[115] Li H, Liu ZY, Wu N, Chen YC, Cheng Q, Wang J. PARP inhibitor resistance: the underlying mechanisms and clinical implications. Mol Cancer. 2020; 19:107. 10.1186/s12943-020-01227-0. 32563252 PMC7305609

[116] Parry D, Guzi T, Shanahan F, Davis N, Prabhavalkar D, Wiswell D, Seghezzi W, Paruch K, Dwyer MP, Doll R, Nomeir A, Windsor W, Fischmann T, et al. Dinaciclib (SCH 727965), a novel and potent cyclin-dependent kinase inhibitor. Mol Cancer Ther. 2010; 9:2344–53. 10.1158/1535-7163.MCT-10-0324. 20663931

[117] Johnson SF, Cruz C, Greifenberg AK, Dust S, Stover DG, Chi D, Primack B, Cao S, Bernhardy AJ, Coulson R, Lazaro JB, Kochupurakkal B, Sun H, et al. CDK12 Inhibition Reverses De Novo and Acquired PARP Inhibitor Resistance in BRCA Wild-Type and Mutated Models of Triple-Negative Breast Cancer. Cell Rep. 2016; 17:2367–81. 10.1016/j.celrep.2016.10.077. 27880910 PMC5176643

[118] Emadi F, Teo T, Rahaman MH, Wang S. CDK12: a potential therapeutic target in cancer. Drug Discov Today. 2020; 25:2257–67. 10.1016/j.drudis.2020.09.035. 33038524

[119] Bono A, La Monica G, Alamia F, Mingoia F, Gentile C, Peri D, Lauria A, Martorana A. In Silico Mixed Ligand/Structure-Based Design of New CDK-1/PARP-1 Dual Inhibitors as Anti-Breast Cancer Agents. Int J Mol Sci. 2023; 24:13769. 10.3390/ijms241813769. 37762072 PMC10531453

[120] Xia Q, Cai Y, Peng R, Wu G, Shi Y, Jiang W. The CDK1 inhibitor RO3306 improves the response of BRCA-proficient breast cancer cells to PARP inhibition. Int J Oncol. 2014; 44:735–44. 10.3892/ijo.2013.2240. 24378347

[121] Christie EL, Pattnaik S, Beach J, Copeland A, Rashoo N, Fereday S, Hendley J, Alsop K, Brady SL, Lamb G, Pandey A, deFazio A, Thorne H, et al. Multiple ABCB1 transcriptional fusions in drug resistant high-grade serous ovarian and breast cancer. Nat Commun. 2019; 10:1295. 10.1038/s41467-019-09312-9. 30894541 PMC6426934

[122] Lawlor D, Martin P, Busschots S, Thery J, O’Leary JJ, Hennessy BT, Stordal B. PARP Inhibitors as P-glyoprotein Substrates. J Pharm Sci. 2014; 103:1913–20. 10.1002/jps.23952. 24700236

[123] Rottenberg S, Jaspers JE, Kersbergen A, van der Burg E, Nygren AO, Zander SA, Derksen PW, de Bruin M, Zevenhoven J, Lau A, Boulter R, Cranston A, O’Connor MJ, et al. High sensitivity of BRCA1-deficient mammary tumors to the PARP inhibitor AZD2281 alone and in combination with platinum drugs. Proc Natl Acad Sci U S A. 2008; 105:17079–84. 10.1073/pnas.0806092105. 18971340 PMC2579381

[124] Hanahan D, Weinberg RA. Hallmarks of cancer: the next generation. Cell. 2011; 144:646–74. 10.1016/j.cell.2011.02.013. 21376230

[125] Hanahan D, Coussens LM. Accessories to the crime: functions of cells recruited to the tumor microenvironment. Cancer Cell. 2012; 21:309–22. 10.1016/j.ccr.2012.02.022. 22439926

[126] Willumsen N, Thomsen LB, Bager CL, Jensen C, Karsdal MA. Quantification of altered tissue turnover in a liquid biopsy: a proposed precision medicine tool to assess chronic inflammation and desmoplasia associated with a pro-cancerous niche and response to immuno-therapeutic anti-tumor modalities. Cancer Immunol Immunother. 2018; 67:1–12. 10.1007/s00262-017-2074-z. 29022089 PMC11028250

[127] Velaei K, Samadi N, Barazvan B, Soleimani Rad J. Tumor microenvironment-mediated chemoresistance in breast cancer. Breast. 2016; 30:92–100. 10.1016/j.breast.2016.09.002. 27668856

[128] Costa A, Kieffer Y, Scholer-Dahirel A, Pelon F, Bourachot B, Cardon M, Sirven P, Magagna I, Fuhrmann L, Bernard C, Bonneau C, Kondratova M, Kuperstein I, et al. Fibroblast Heterogeneity and Immunosuppressive Environment in Human Breast Cancer. Cancer Cell. 2018; 33:463–79.e10. 10.1016/j.ccell.2018.01.011. 29455927

[129] Busch S, Acar A, Magnusson Y, Gregersson P, Rydén L, Landberg G. TGF-beta receptor type-2 expression in cancer-associated fibroblasts regulates breast cancer cell growth and survival and is a prognostic marker in pre-menopausal breast cancer. Oncogene. 2015; 34:27–38. 10.1038/onc.2013.527. 24336330

[130] Pickup MW, Mouw JK, Weaver VM. The extracellular matrix modulates the hallmarks of cancer. EMBO Rep. 2014; 15:1243–53. 10.15252/embr.201439246. 25381661 PMC4264927

[131] Lin L, Chen YS, Yao YD, Chen JQ, Chen JN, Huang SY, Zeng YJ, Yao HR, Zeng SH, Fu YS, Song EW. CCL18 from tumor-associated macrophages promotes angiogenesis in breast cancer. Oncotarget. 2015; 6:34758–73. 10.18632/oncotarget.5325. 26416449 PMC4741488

[132] Wang L, Wang D, Sonzogni O, Ke S, Wang Q, Thavamani A, Batalini F, Stopka SA, Regan MS, Vandal S, Tian S, Pinto J, Cyr AM, et al. PARP-inhibition reprograms macrophages toward an anti-tumor phenotype. Cell Rep. 2022; 41:111462. 10.1016/j.celrep.2022.111462. 36223740 PMC9727835

[133] Ding L, Wang Q, Martincuks A, Kearns MJ, Jiang T, Lin Z, Cheng X, Qian C, Xie S, Kim HJ, Launonen IM, Färkkilä A, Roberts TM, et al. STING agonism overcomes STAT3-mediated immunosuppression and adaptive resistance to PARP inhibition in ovarian cancer. J Immunother Cancer. 2023; 11:e005627. 10.1136/jitc-2022-005627. 36609487 PMC9827255

[134] Vadde R, Vemula S, Jinka R, Merchant N, Bramhachari PV, Nagaraju GP. Role of hypoxia-inducible factors (HIF) in the maintenance of stemness and malignancy of colorectal cancer. Crit Rev Oncol Hematol. 2017; 113:22–7. 10.1016/j.critrevonc.2017.02.025. 28427511

[135] De Palma M, Biziato D, Petrova TV. Microenvironmental regulation of tumour angiogenesis. Nat Rev Cancer. 2017; 17:457–74. 10.1038/nrc.2017.51. 28706266

[136] Fertig EJ, Lee E, Pandey NB, Popel AS. Analysis of gene expression of secreted factors associated with breast cancer metastases in breast cancer subtypes. Sci Rep. 2015; 5:12133. 10.1038/srep12133. 26173622 PMC4648401

[137] Tsuyada A, Chow A, Wu J, Somlo G, Chu P, Loera S, Luu T, Li AX, Wu X, Ye W, Chen S, Zhou W, Yu Y, et al. CCL2 mediates cross-talk between cancer cells and stromal fibroblasts that regulates breast cancer stem cells. Cancer Res. 2012; 72:2768–79. 10.1158/0008-5472.CAN-11-3567. 22472119 PMC3367125

[138] Murai T. Lipid Raft-Mediated Regulation of Hyaluronan-CD44 Interactions in Inflammation and Cancer. Front Immunol. 2015; 6:420. 10.3389/fimmu.2015.00420. 26347743 PMC4542320

[139] Dzobo K, Dandara C. The Extracellular Matrix: Its Composition, Function, Remodeling, and Role in Tumorigenesis. Biomimetics (Basel). 2023; 8:146. 10.3390/biomimetics8020146. 37092398 PMC10123695

[140] Inoue T, Sekito S, Kageyama T, Sugino Y, Sasaki T. Roles of the PARP Inhibitor in *BRCA1* and *BRCA2* Pathogenic Mutated Metastatic Prostate Cancer: Direct Functions and Modification of the Tumor Microenvironment. Cancers (Basel). 2023; 15:2662. 10.3390/cancers15092662. 37174127 PMC10177034

